# A Review of Aggregation-Based Colorimetric and SERS Sensing of Metal Ions Utilizing Au/Ag Nanoparticles

**DOI:** 10.3390/bios16020110

**Published:** 2026-02-08

**Authors:** Shu Wang, Lin Yin, Yanlong Meng, Han Gao, Yuhan Fu, Jihui Hu, Chunlian Zhan

**Affiliations:** 1 College of Metrology Measurement and Instrument, China Jiliang University, Hangzhou 310018, China; b24020804018@cjlu.edu.cn; 2 College of Optical and Electronic Technology, China Jiliang University, Hangzhou 310018, China; myl@cjlu.edu.cn (Y.M.); gaohan@cjlu.edu.cn (H.G.); p24040854184@cjlu.edu.cn (Y.F.); s24040809007@cjlu.edu.cn (J.H.)

**Keywords:** Au/Ag nanoparticles, surface-enhanced Raman scattering (SERS), colorimetric sensing, metal ions, aggregation, localized surface plasmon resonance (LSPR)

## Abstract

The accurate monitoring and dynamic analysis of metal ions are of considerable practical significance in environmental toxicology and life sciences. Colorimetric analysis and surface-enhanced Raman scattering (SERS) sensing technologies, utilizing the aggregation effect of gold and silver nanoparticles (Au/Ag NPs), have emerged as prominent methods for rapid metal ion detection. While sharing a common plasmonic basis, these two techniques serve distinct yet complementary analytical roles: colorimetric assays offer rapid, instrument-free visual screening ideal for point-of-care testing (POCT), whereas SERS provides superior sensitivity and structural fingerprinting for precise quantification in complex matrices. Furthermore, the synergistic integration of these modalities facilitates the development of dual-mode sensing platforms, enabling mutual signal verification for enhanced reliability. This article evaluates contemporary optical sensing methodologies utilizing aggregation effects and their advancements in the detection of diverse metal ions. It comprehensively outlines methodological advancements from nanomaterial fabrication to signal transduction, encompassing approaches such as biomass-mediated green synthesis and functionalization, targeted surface ligand engineering, digital readout systems utilizing intelligent algorithms, and multimodal synergistic sensing. Recent studies demonstrate that these techniques have attained trace-level identification of target ions regarding analytical efficacy, with detection limits generally conforming to or beyond applicable environmental and health safety regulations. Moreover, pertinent research has enhanced detection linear ranges, anti-interference properties, and adaptability for POCT, validating the usefulness and developmental prospects of this technology for analysis in complicated matrices.

## 1. Introduction

Metal ions are omnipresent in the environment, prevalent in natural water bodies, soil matrices, and biological fluids. The real-time monitoring and accurate analysis of their concentrations are crucial for preserving global ecological balance, ensuring food safety, and enabling early clinical diagnosis [1]. In environmental toxicology, many heavy metal pollutants, including chromium (Cr^3+^, Cr^6+^), mercury (Hg^2+^), and lead (Pb^2+^), exhibit significant environmental persistence and bioaccumulation potential. Even at trace concentrations, these pollutants can bioaccumulate within the food chain, resulting in irreparable harm to the central nervous system and other essential organs [2,3]. In contrast, transition metal ions, such as copper (Cu^2+^) and iron (Fe^2+^, Fe^3+^), are crucial for vital biological functions; nonetheless, deviations from normal physiological concentrations—either deficiency or excess—can lead to oxidative stress or cytotoxicity through the Fenton reaction. This imbalance is significantly associated with neurodegenerative disorders, such as Parkinson’s disease [4,5]. Moreover, the ecological behavior and biological consequences of alkali metals (e.g., Na^+^) related to osmotic control [6], potentially hazardous aluminum ions (Al^3+^) [7], and emerging rare earth elements [8] necessitate thorough examination.

Traditionally, extensive instrumental analysis methods—such as inductively coupled plasma mass spectrometry (ICP-MS) [9,10], inductively coupled plasma optical emission spectrometry (ICP-OES) [11], and atomic absorption spectroscopy (AAS)—have been considered the “gold standard” for the quantitative assessment of metal ions [12]. These techniques are distinguished by their exceptionally low detection limits, extensive linear ranges, and superior reproducibility. Nonetheless, their extensive applicability is significantly restricted by intrinsic limits, such as costly instrumentation, substantial size, elevated operational and maintenance expenses, and stringent sample pretreatment prerequisites (e.g., acid digestion). These limitations substantially impede their implementation for real-time monitoring in resource-constrained areas or during abrupt environmental pollution incidents. To overcome the limitations of conventional techniques, researchers have increasingly focused on creating more efficient and expedited detection methods. As a result, other innovative methodologies have arisen, including electrochemical analysis [13,14], fluorescent probe technology [15,16], surface-enhanced Raman scattering (SERS) [17], and colorimetric analysis [18]. These methods maintain excellent detection sensitivity while markedly decreasing reliance on extensive equipment, thereby offering viable options for the swift on-site detection of metal ions.

Among these optical methodologies, colorimetric analysis and SERS sensing employing noble metal nanoparticles (e.g., Au, Ag) are particularly significant due to their common dependence on localized surface plasmon resonance (LSPR). The rationale for focusing on these two procedures lies in their individual analytical strengths and their potential for synergistic integration. While colorimetric assays offer rapid, instrument-free visual screening ideal for point-of-care testing (POCT) [18], and SERS provides superior sensitivity and structural fingerprinting for precise quantification in complex matrices [17], their combination into dual-mode platforms enables mutual signal verification, effectively bridging the gap between on-site convenience and analytical reliability.

The core premise of colorimetric sensing entails converting the interaction between metal ions and nanoprobes into observable spectrum shifts or discernible color alterations. This technique is highly advantageous for POCT applications where simplicity and cost-effectiveness are paramount. Contemporary construction methodologies involve morphology etching, dissolution, or amalgamation through the redox characteristics of target ions [19,20,21]; the generation of precipitates via specific recognition adsorption or in situ reactions between probe molecules and ions [22,23]; and ion-induced nanoparticle aggregation. Aggregation-based colorimetric detection is a crucial method for facilitating swift ion analysis. This technique utilizes metal ions to diminish inter-particle distances, hence inducing strong dipole–dipole interactions and plasmonic coupling phenomena. The physical coupling results in a notable red shift and broadening of the LSPR absorption band, which is macroscopically observed as a change in solution color, thus offering dual modalities for instrumental spectral analysis and on-site visual detection of metal ions [24,25].

While colorimetry delivers accessible optical feedback for qualitative or semi-quantitative screening, SERS technology with analogous nanostructured substrates adds an additional detection dimension for enhanced fingerprint identification and rigorous quantitative analysis of trace analytes in complicated matrices. Due to the limited Raman scattering cross-sections of most metal ions, SERS detection often depends on indirect sensing frameworks. Recent studies have established several sophisticated mechanisms, including signal amplification techniques leveraging aptamer-regulated nanozyme catalytic activity [26], recognition approaches employing generic ligands to interact with multiple metal ions for distinct spectral fingerprints [27,28], ratiometric sensing reliant on conformational switches of DNA nanostructures [29], and “signal-off” mechanisms predicated on competitive ligand desorption [30,31]. In addition to these ways, metal ion-induced controlled aggregation functions as an efficient building method. This method adjusts the density of electromagnetic “hotspots” by modifying the aggregation state of nanoparticles, thus controlling the enhancement factor (EF) to create “signal-on” or “signal-off” sensors. The sub-nanometer gaps created by dense packing in aggregates can produce an exponentially increasing local electromagnetic field, greatly enhancing the signal of Raman reporter molecules situated at the hotspots; in contrast, the disruption of these aggregated structures results in the loss of hotspots and signal attenuation. This exact manipulation of the “aggregation–hotspot–signal intensity” cascade process by physical coupling offers a high signal-to-noise ratio detection platform for trace metal ion analysis [32].

## 2. Fundamentals of Aggregation-Based Detection

The identification of metal ions through Au/Ag nanoparticle aggregation involves four specific stages: nanomaterial synthesis, ion-interface interaction, optical signal transmission, and data processing. Gold or silver nanoparticles (Au/Ag NPs) with defined morphologies and consistent dimensions are often generated as substrates using chemical reduction or seed-mediated growth techniques [33,34,35]. The introduction of target metal ions induces specific physicochemical interactions with the surface ligands or electric double layers of the nanoparticles, disrupting the metastable equilibrium of the colloidal system and prompting the transition of particles from a dispersed to an aggregated state. This aggregation tendency significantly modifies the LSPR properties of the system. This is observed macroscopically as a shift and broadening of the absorption spectra, representing the colorimetric response [18,36]. At the microscopic level, the decrease in inter-particle distance promotes the creation of “hotspots” characterized by elevated electromagnetic field density within the interstices, leading to a significant amplification of SERS signals [32,37]. These optical responses are finally recorded by spectral equipment or portable devices for the qualitative identification and quantitative study of target ions [38].

The mechanisms by which metal ions induce nanoparticle aggregation primarily encompass three categories of physicochemical processes. The primary factor is the reduction in electrostatic repulsion and the compression of the electric double layer. The introduction of exogenous metal ions elevates the ionic strength of the solution, compressing the electric double layer on the particle surface and screening surface charges, which facilitates van der Waals attraction to prevail and induce aggregation [39,40,41]. The second mechanism is coordination bridging-induced assembly. Metal ions serve as coordination centers by binding to functional ligands comprising sulfur, nitrogen, or oxygen modified on the particle surface, thereby creating a permanent bridging network that facilitates the proximity of particles [42,43]. The third mechanism is instability driven by oxidative etching. This technique utilizes the elevated redox potential of particular metal ions to facilitate the oxidation and partial dissolution of the nanoparticle surface or the degradation of stabilizing ligands. The etching impact undermines the integrity of the surface protective layer and diminishes surface potential, thereby compromising colloidal stability and inducing irreversible aggregation [44,45,46].

Recent methodological advancements in colorimetric detection span seven principal dimensions. (1) Green synthesis strategies utilizing biomass employ natural plant extracts or biomass derivatives to act as reducing agents, stabilizers, and recognition ligands, thereby streamlining the synthesis process and incorporating inherent anti-interference properties. (2) Ligand engineering encompasses not just individual ligands but also the design of synthetic small molecules, the use of biological macromolecules like DNA or enzymes, and the creation of dual-ligand synergistic systems to facilitate the exact capture and interfacial manipulation of specific ions. (3) Special Response Mechanisms overcome the constraints of conventional electrostatically generated aggregation by employing several methods, such as metal ion-mediated lattice doping, oxidative etching, anti-aggregation kinetic regulation, and ligand-metal charge transfer (LMCT). (4) Physical Assistance and Post-Treatment methods incorporate microwave or laser liquid ablation during the synthesis phase to improve reaction speeds and surface cleanliness, while employing pH-selective precipitation in the post-treatment phase to enhance colloidal purity. (5) Solid-Phase Support and Phase Transition tackle the difficulties of storing and transporting liquid-phase colloids by implementing hydrogel three-dimensional networks or paper-based microfluidic chips, hence broadening the application contexts for Point-of-Care Testing (POCT). (6) Multimodal sensing develops dual-mode sensing systems that utilize light/light (e.g., fluorescence-colorimetric) or light/electric mechanisms, while also incorporating catalytic degradation functions to achieve interactive signal verification and the integration of “diagnosis and therapy.” (7) Smart Readout and Algorithmic Enhancement integrate smartphone photography technology with machine learning algorithms, including Random Forest and Support Vector Machines (SVMs), to facilitate digital calibration and accurate quantification of signals in intricate lighting conditions.

The methodological approach for SERS detection encompasses five key factors. (1) Molecular Probe Engineering creates “beacons” by incorporating dyes or functional molecules with elevated Raman scattering cross-sections, formulating bifunctional molecular approaches that merge chelation with luminescence, alongside multi-component synergistic modification strategies to tackle the challenge of metal ions devoid of intrinsic Raman signals. (2) Plasmonic nanohybrid design meticulously organizes the spatial distribution and stability of electromagnetic hotspots at the nanoscale by the fabrication of Au@Ag core–shell structures, core-satellite assemblies, or magnetic composites. (3) Special Response Mechanisms employ dynamic chemical processes—such as metal ion-catalyzed Fenton-like reactions, DNAzyme cleavage, oxidative etching, or spintronic effects—to regulate the “signal-on” and “signal-off” phases of spectrum signals. Moreover, SERS research predominantly employs (4) Green synthesis strategies utilizing biomass substrates akin to those utilized in colorimetry to improve sustainability. (5) Chemometrics-Assisted Analysis focuses on high-dimensional spectral data by employing genetic algorithms (GAs) or partial least squares (PLS) to create predictive models, thereby addressing issues associated with non-linear signal responses during aggregation and interference from intricate matrix backgrounds.

Furthermore, recent advancements have increasingly focused on integrating these optical modalities into dual-mode sensing platforms. By synergizing the rapid visual feedback of colorimetry with the structural fingerprinting of SERS, these systems enable mutual signal verification and self-calibration, effectively mitigating false positives in complex analytical scenarios.

**Figure 1:**
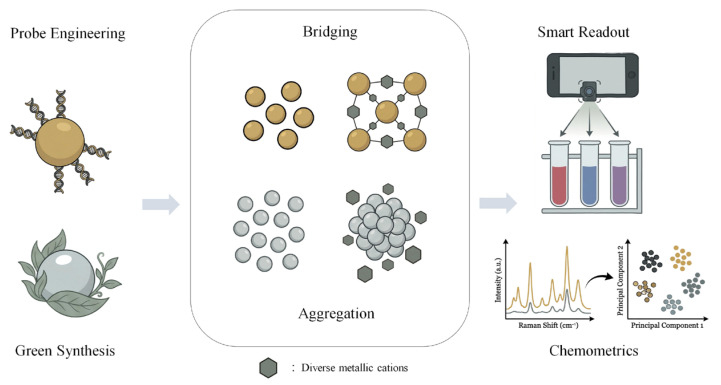
Schematic depiction of aggregation-based colorimetric and SERS sensing techniques for metal ions.

Figure 1 delineates a roadmap of recent achievements, summarizing the principal methodologies mentioned, and displaying the workflow from nanomaterial fabrication and mechanism manipulation to intelligent analytical readouts.

The varied advancements in synthesis methodologies, interfacial chemistry, and signal transduction processes mentioned above are primarily intended to exceed current detection limitations and improve the practicality and reliability of analytical procedures. The examined study exhibits excellent selectivity, accuracy, and reliability; nonetheless, they vary in the specific performance criteria prioritized for practical applications. This review utilizes unique tags to annotate pertinent material, thus elucidating the distinct qualities of each contribution. In the realm of metal ion detection, “Ultrasensitive” refers to research that presents the lowest limit of detection (LOD), signifying a comparative benefit in trace analysis; “Wide linear range” implies studies that attain the most extensive linear response range among analogous assays. Concerning functional attributes, “Sensitive & Portable” denotes research that adeptly reconciles high sensitivity with on-site portability; “Self-calibrated” refers to systems equipped with signal self-calibration capabilities, effectively reducing environmental background interference to guarantee high reliability. Moreover, “High-stable” refers to research in which probes have been empirically validated to demonstrate considerable long-term storage stability.

## 3. Colorimetric Sensors

### 3.1. Heavy Metal Ions

This section addresses the colorimetric detection of heavy metal elements, specifically chromium (Cr), mercury (Hg), and lead (Pb). The analytical efficacy of these colorimetric tests for identifying chromium ions (Cr^3+^ and Cr^6+^) is summarized in Table 1. Correspondingly, the analytical efficacy for the identification of mercury (Hg) and lead (Pb) ions is presented in Table 2.

**Figure 2:**
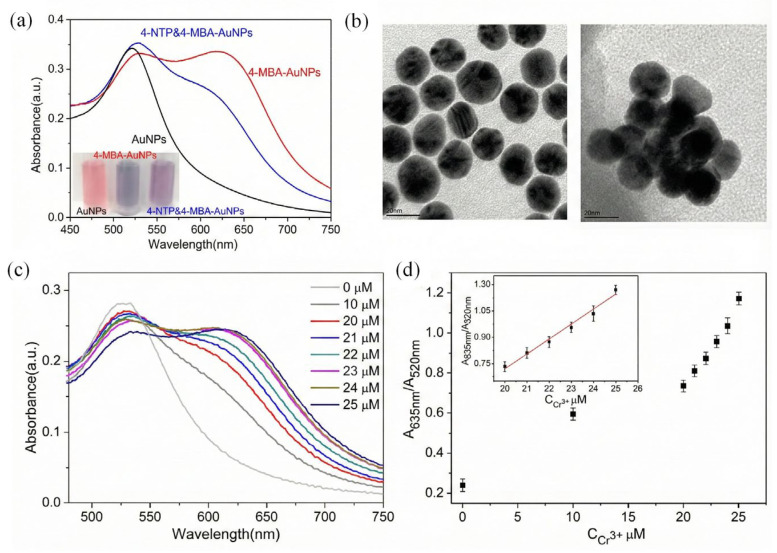
Evaluation and characterisation of the 4-MBA-AuNPs probe for the detection of Cr^3+^. (**a**) Comparison of UV-vis absorption spectra for AuNPs modified with different ligands in the presence of Cr^3+^; inset shows the corresponding visual color changes. (**b**) TEM images illustrating 4-MBA-AuNPs in the absence (**left**) and presence (**right**) of Cr^3+^, indicating induced aggregation. (**c**) Evolution of the absorption spectra with increasing Cr^3+^ concentrations (0–25 μM). (**d**) Linear calibration curve plotting the absorbance ratio (A_635_/A_520_) versus Cr^3+^ concentration [47].

Trivalent chromium (Cr^3+^) is a critical subject of environmental monitoring owing to its potential detrimental impacts on human health and ecological systems at high concentrations. Zhang et al. [47] extensively evaluated the efficacy of several surface ligands in their response to Cr^3+^. The researchers developed a colorimetric probe utilizing 4-mercaptobenzoic acid (4-MBA) functionalized AuNPs and conducted a comparison with a 4-nitrothiophenol (4-NTP) device. Spectral analysis demonstrated that 4-MBA-modified AuNPs had the most pronounced absorbance variation and best sensitivity (Figure 2a). The detection method is attributed to ion-templated chelation, in which Cr^3+^ serves as a coordination center that precisely binds to the carboxyl groups on the AuNP surface. TEM imaging clearly demonstrated a transition of the nanoparticles from a monodispersed state to discrete aggregates upon the introduction of ions (Figure 2b), leading to a color change in the solution from wine-red to purple or blue. As the concentration of Cr^3+^ increased, the LSPR absorption band of the 4-MBA-functionalized AuNPs exhibited a consistent red shift (Figure 2c). The absorbance ratio (A_635 nm_/A_520 nm_) exhibited a strong linear correlation in the range of 20–25 μM (Figure 2d), with a LOD of 5 μM.

The use of natural biomass for nanomaterial production offers a cost-effective method for detection. Memon et al. [48] utilized Ziziphus mauritiana leaf (ZmL) extract, abundant in carbonyl groups, as a reducing and capping agent to synthesize ZmL-AuNPs. The presence of Cr^3+^ dramatically enhanced the average size of the ZmL-AuNPs from 8 nm to 25 nm. This biosynthesized sensor attained a remarkably low limit of detection of 0.48 nM, with a linear range of 16–283 nM, and exhibited exceptional stability in complicated aqueous samples.

To address the performance constraints of individual nanomaterials, techniques using hybrid materials and multifunctional ligands have been developed. Shellaiah and Sun [49] ingeniously developed a hybrid probe that integrates cystamine-functionalized nanodiamonds (NDC) with AuNPs (NDC-AuNPs). This study utilized the extensive specific surface area and superior biocompatibility of NDCs as a carrier, facilitating aggregation through the coordination of surface amino/thiol groups with Cr^3+^. This approach attained a sub-nanomolar limit of detection (0.236 nM) and a linear range of 10–400 nM, demonstrating commendable cyclic reversibility post-EDTA treatment.

**Figure 3:**
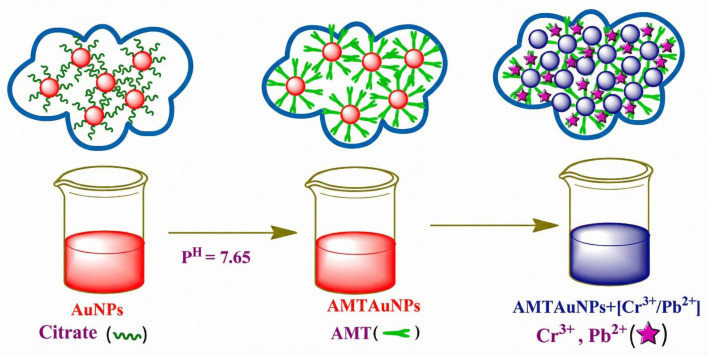
Schematic representation of the synthesis of AMT-functionalized AuNPs and the colorimetric detection technique for Cr^3+^ and Pb^2+^ [50].

Gunupuru and Paul [50] utilized amino-substituted 2-amino-5-mercapto-1,3,4-thiadiazole (AMT) to alter AuNPs, thereby creating a dual-functional colorimetric sensing platform. This method initially tethered AMT to the surface of gold nanoparticles by thiol groups, as depicted in Figure 3. In the presence of target ions, Cr^3+^ and Pb^2+^ interacted with surface amino groups, resulting in significant nanoparticle aggregation. This led to a redshift of the LSPR band and a color change from wine-red to blue. The approach attained limits of detection of 1.0 µM for Cr^3+^ and 0.4 µM for Pb^2+^ in the aqueous phase.

Zhang et al. [51] developed a multifunctional platform utilizing ammonium thioglycolate (ATG) functionalized AuNPs. By employing various active sites (carboxyl groups and fluorine atoms) on the ATG surface, they accomplished the concurrent screening of moxifloxacin, ciprofloxacin, and Cr^3+^. This platform displayed a limit of detection of 57.1 nM for Cr^3+^ with a linear response range of 0–5.0 µM, illustrating the adaptability of functionalized probes in the assessment of multi-component contaminants.

To overcome the constraints of single-signal detection, Shi et al. [52] devised a dual-mode fluorescence/colorimetric sensing system utilizing carbon dots (CDs) and GSH-Au nanoparticles. The inner filter effect (IFE) facilitated Cr^3+^-induced aggregation, producing a colorimetric signal while concurrently restoring the fluorescence of the CDs in a “turn-on” mode. At pH 5.4, the limits of detection for the fluorescence and colorimetric modalities were 0.31 μM and 0.30 μM, respectively, with linear ranges of 0.5–70 μM and 2–50 μM. This dual-mode cross-verification approach markedly improved data reliability in intricate matrices.

Algorithms embedded in smartphones have become essential for swift on-site detection. Rajamanikandan et al. [53] created a portable platform integrated with smartphone RGB analysis. This platform utilized the significant reduction in surface potential (from −33.12 mV to −5.10 mV) resulting from the coordination of Cr^3+^ with MMT ligands to mitigate electrostatic repulsion—attaining an LOD of 12.4 nM (spectrophotometric LOD 6.93 nM, linear range 40–128 nM)—while also incorporating catalytic degradation capabilities, thereby preliminarily actualizing the concept of “theranostics” (diagnosis and therapy). To further enhance the integration and automation of portable on-site detection, Moradifar et al. [54] developed a smartphone-based microfluidic colorimetric kit utilizing polymethyl methacrylate (PMMA) for point-of-care (POC) applications. The researchers methodically enhanced flow rate and pH parameters through a central composite design (CCD) model, addressing the problem of inconsistent mixing in conventional liquid-phase detection and minimizing reagent usage. The platform demonstrated a favorable linear response within the range of 1.00–35.00 µM, with a limit of detection of 0.33 µM, thereby affirming the practical utility of microfluidic technology for high-throughput on-site screening.

Hexavalent chromium (Cr^6+^) is a primary focus in water monitoring because of its significant carcinogenic properties and environmental durability. Sharma et al. [55] employed a microwave-assisted technique to swiftly synthesize chlorophyll-coated silver nanoparticles (Chl-AgNPs) in 10 s. This study utilized natural chlorophyll as a reducing and capping agent, leveraging its rich surface functional groups (such as methyl and carboxyl groups) as capture sites for Cr^6+^ to produce alterations in interparticle distance and aggregation. This was spectroscopically indicated by a red shift of the 410 nm characteristic peak and the appearance of a new peak at 357 nm. The sensor demonstrated excellent linearity throughout the 2–100 μM range (LOD 0.62 μM), and the distinct red color development effectively mitigated interference from As^5+^. Skiba et al. [56] investigated the gas-liquid interfacial plasma discharge technique as an additional physical auxiliary strategy, successfully facilitating the one-pot fast synthesis of PVP-stabilized gold nanoparticles. This approach, leveraging the abundant active species and electron transfer produced during the plasma process, achieved synthesis within minutes without conventional reducing agents, guaranteeing exceptional surface purity of the nanoparticles. The study indicated a significant reliance of detection performance on pH: in acidic environments, Cr^6+^ predominantly exists as HCrO_4_^−^, resulting in strong electrostatic attraction to the protonated PVP surface and causing substantial aggregation; in contrast, under alkaline conditions, the electrostatic repulsion from the CrO_4_^2−^ form impedes the signal. The revised mechanism enabled the sensor to provide a superior linear response within the range of 0.1–3.0 µM, with a limit of detection of 0.072 µM.

Muthwa et al. [57] adeptly integrated experimental characterisation with molecular dynamics (MD) simulations to elucidate ligand-ion interactions in a 1,5-diphenylcarbazide (DPC) functionalized gold nanoparticle system. The radial distribution function (RDF) study in the molecular dynamics simulation verified a significant attraction between Cr^6+^ and the nitrogen atoms in DPC, with a contact distance of around 3 Å. This interaction was markedly more potent than the binding force between DPC and the Au surface, resulting in ligand detachment from the nanoparticle surface. The AuNPs, shedding their protective coating, swiftly experienced uncontrolled aggregation, resulting in a color change in the solution from wine-red to blue. The LSPR peak exhibited a red shift from 520 nm and a reduction in intensity, while a new peak emerged at 670 nm. This approach attained a low limit of detection of 0.3 μM and incorporated smartphone-based CIE Lab* color space analysis, including hue angle and chroma. This method, in contrast to conventional absorbance techniques, yielded more comprehensive fingerprint data, hence enhancing theoretical insights into ligand-mediated aggregation behavior.

**Figure 4:**
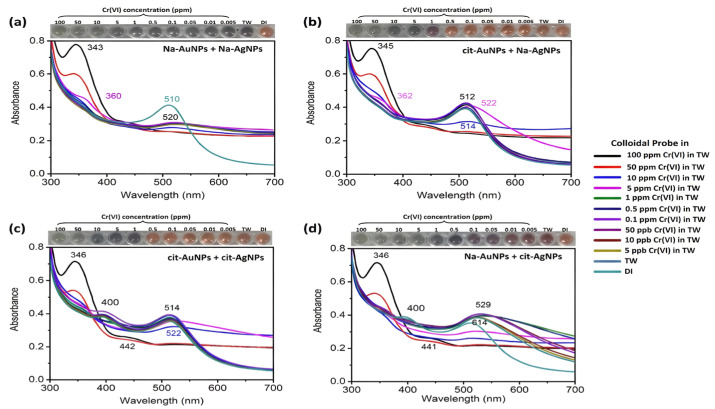
Comparative analysis of extinction spectra and perceptual color alterations in AuNP/AgNP mixes with varying capping agents following the addition of Cr^6+^. (**a**) Na-AuNPs/Na-AgNPs, (**b**) cit-AuNPs/Na-AgNPs, (**c**) cit-AuNPs/cit-AgNPs, and (**d**) Na-AuNPs/cit-AgNPs [58].

The elevated redox potential of Cr^6+^ can be further utilized to improve visual resolution. Karn-orachai et al. [58] conducted a comprehensive evaluation of the impact of various combinations of capping agents on probe performance (Figure 4). The comparative results indicated that only the bimetallic sol system, which combines Na-AuNPs and cit-AgNPs (Figure 4d), could facilitate a synergistic “oxidative etching-aggregation” mechanism. The potent Cr^6+^ initially oxidatively etched the AgNPs and AuNPs, resulting in the release of metal ions that caused significant aggregation and redeposition of the residual particles. This intricate chemical-physical process imparted the sensor with a spectrum of vivid color transitions—progressing from orange to deep reddish-purple, then to deep bluish-purple, and finally to gray as concentration escalated—significantly improving the resolution of unaided semi-quantitative analysis. The approach attained a LOD of roughly 0.44 μM (22.9 ppb) with a linear range of 0.05–50 ppm, showcasing distinct benefits in anti-interference efficacy and visual clarity.

Mercury ions (Hg^2+^), as highly toxic heavy metal contaminants, present significant risks to ecological systems and human health, making their trace detection very important. The fundamental recognition process of colorimetric sensing for Hg^2+^ predominantly follows the “reduction-amalgamation-aggregation” principle. Esquivel Rincón et al. [59] introduced a physical synthesis approach utilizing Laser Ablation Synthesis in Solution (LASiS), successfully avoiding surface contamination from chemical reducing agent residues present in conventional procedures. The team effectively synthesized high-purity AgNPs exhibiting a face-centered cubic (FCC) structure and clarified a distinctive “reduction-amalgamation-aggregation” sensing mechanism: Hg^2+^ is initially reduced to Hg^0^ with the aid of sodium citrate, subsequently permeating the AgNP lattice to create an Ag/Hg amalgam. STEM elemental mapping verified the spatial co-localization of Ag and Hg, whereas a slight alteration in Zeta potential (from −27.12 mV to −25.31 mV) resulted in considerable particle aggregation (particle size rose to 167 nm). This system demonstrated a significant reduction in LSPR peak strength at 400 nm, accompanied by a wavelength blueshift, resulting in a visual change from bright yellow to colorless, with a detection limit of 0.2 ppm.

Biomass-mediated synthesis methodologies have attracted considerable interest owing to their cost-effectiveness and ecological sustainability; nevertheless, the disparate redox potentials of various extracts markedly affect the sensing processes. Tewari et al. [60] employed tannin-rich Diospyros kaki leaf extract to synthesize AgNPs. Their postulated mechanism suggested that the nanoscale size effect diminished the redox potential of the AgNPs, facilitating the reduction and deposition of Hg^2+^ to create an amalgam, which led to an LSPR blueshift and fluorescence quenching. This probe exhibited exceptional sensitivity (LOD 0.1 ppb) and an extensive linear range (0.1–100,000 ppb), and was effectively utilized for fluorescence imaging of liver tissue cells and pathogen suppression owing to its superior biocompatibility. Conversely, Thepwat and Kosolwattana [61] utilized carboxymethyl cellulose (CMC) derived from water hyacinth as a bifunctional compound. Due to the disparity in standard potentials (Hg^2+^/Hg^0^ +0.85 V versus Ag^+^/Ag^0^ +0.80 V), Hg^2+^ oxidized the surface Ag^0^ of the AgNPs while undergoing reduction to create an amalgam. This method eradicated electrostatic repulsion, resulting in a substantial increase in Zeta potential from −26.2 mV to −5.89 mV, hence inducing significant aggregation. Despite its detection limit of 3.14 μM being marginally greater than that documented by Tewari, the probe exhibited remarkable anti-interference properties in complex aqueous matrices (e.g., containing Ca^2+^, Mg^2+^) and displayed a robust linear correlation within the concentration range of 5–45 µM. Mume et al. [62] further developed this strategy employing Salvia tiliifolia extract, attaining highly sensitive detection of Hg^2+^ (LOD 0.27 nM, linear range 0.1–100 µM) through a comparable amalgamation-induced irregular aggregation mechanism, which was effectively utilized for the analysis of tuna and environmental water samples.

**Figure 5:**
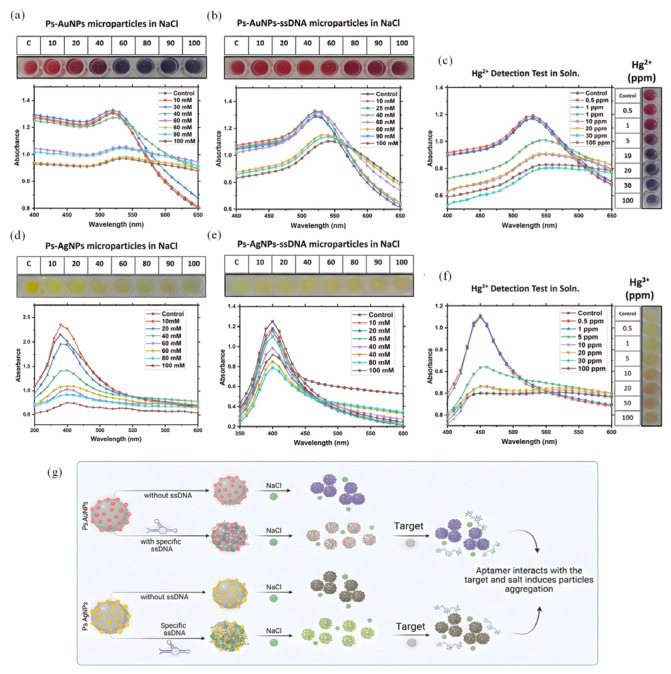
Evaluation of stability and colorimetric sensing efficacy of the aptamer-functionalized microparticles. (**a**) Salt-induced aggregation of bare Ps-AuNPs at different NaCl concentrations. (**b**) Stability test of aptamer-functionalized Ps-AuNPs (Ps-AuNPs-ssDNA) in NaCl solutions. (**c**) UV-vis spectra and colorimetric response of Ps-AuNPs-ssDNA to varying Hg^2+^ concentrations. (**d**) Salt-induced aggregation of bare Ps-AgNPs at different NaCl concentrations. (**e**) Stability test of aptamer-functionalized Ps-AgNPs (Ps-AgNPs-ssDNA) in NaCl solutions. (**f**) UV-vis spectra and colorimetric response of Ps-AgNPs-ssDNA to varying Hg^2+^ concentrations. (**g**) Schematic illustration of the sensing mechanism based on aptamer-target binding and particle aggregation [63].

In addition to the previously indicated physicochemical methods, employing the particular coordination of nucleic acids constitutes another essential strategy. Ulloa-Gomez et al. [63] created a dual-mode aptasensor that combines microfluidic paper-based analytical devices (μ-PADs) with miniature printed circuit boards (PCBs) for swift on-site trace detection. The sensing process predominantly depends on the establishment of the particular T-Hg^2+^-T mismatch structure. Figure 5g illustrates that this method facilitates signal transmission through a salt-mediated aggregation process. Stability assessments validated that the incorporation of ssDNA aptamers proficiently protected the electric double layer of the nanoparticles from compression due to elevated ionic strength, thereby preserving probe dispersion in saline solutions (Figure 5a,b,d,e); however, the presence of Hg^2+^ induced the aptamer to adopt a rigid duplex conformation and dissociate from the particle surface, consequently eliciting an aggregation response. The alteration in microscopic state within the colorimetric module led to a notable shift or intensity reduction in the LSPR absorption peak (Figure 5c,f), resulting in a discernible color change. Concerning analytical performance, the polystyrene (Ps)-AgNPs-based colorimetric system attained a linear detection range of 0.5–20 ppm with a LOD of 0.5 ppm, whereas the Ps-AuNPs variant was mostly utilized for qualitative screening (LOD 5 ppm). The electrochemical module demonstrated superior sensitivity, achieving a limit of detection of 0.01 ppm.

Yang et al. [64] devised a label-free colorimetric sensing approach utilizing a DNA-mediated charge neutralization mechanism of AuNPs, aimed at the on-site detection of Hg^2+^ in environmental water and cosmetics. This research developed a “probe-blocker” double-stranded DNA system consisting of probe DNA (p-DNA) and blocker DNA (b-DNA). The sensing mechanism utilizes the strong affinity of T-Hg^2+^-T specific mismatched base pairs: in the presence of the target Hg^2+^, the replacement DNA (r-DNA) preferentially associates with the p-DNA, resulting in the release of the long-chain b-DNA from the double-stranded configuration. The free b-DNA, owing to its extended chain length, efficiently adsorbs onto the AuNP surface, thereby markedly impeding Thioflavin T (ThT)-induced charge neutralization and aggregation of the AuNPs. This approach accomplished visual detection of Hg^2+^ using smartphone image analysis of the chromaticity alteration of the AuNP solution from blue (aggregated state) to red (dispersed state), without necessitating supplementary enzymatic aid or signal amplification procedures. Experimental data indicated that this approach had a linear response range for Hg^2+^ of 0.005–1 μM, with a LOD of 2.85 nM. This sensor platform integrates portable smartphone data gathering technology, offering a cost-effective, high-precision analytical instrument for the swift on-site detection of detectable mercury contamination in the environment and daily consumer products.

To overcome the restrictions of high cost and sensitivity to deactivation associated with biological macromolecules, Liu et al. [65] broadened the sensing application of pharmaceutical small molecules by building a ribavirin-functionalized gold nanoparticle probe (Rib-AuNPs). This method employs electrostatic interactions to attach positively charged ribavirin to the surface of negatively charged AuNPs. DFT theoretical simulations and Electrostatic Potential (ESP) research demonstrated that the triazole nitrogen atoms and amide group oxygen atoms on the ribavirin surface function as highly reactive sites, capable of establishing a stable “chelating-bridging” structure with Hg^2+^. This particular coordination diminishes the electrostatic repulsion among nanoparticles (Zeta potential reduced from −31.7 mV to −18.2 mV), prompting a notable aggregation transition of AuNPs from wine-red to gray-blue (LSPR peak redshifted from 520 nm to 654 nm). This sensor exhibited remarkable anti-interference capability in intricate matrices like tap water and lake water (withstanding 16 competing ions) and accomplished segmented detection across an extensive linear range. The UV-vis spectral limit of detection was 3.64 nM, while the naked-eye visual detection limit was 0.20 mM, offering a quick and highly selective analytical instrument for environmental water monitoring.

The trace accumulation of lead ions (Pb^2+^), a non-essential and highly neurotoxic heavy metal, in environmental water bodies poses a severe challenge to public health. In the realm of green synthesis of nanomaterials, Do Dat et al. [66] generated AuNPs via a one-step approach utilizing Andrographis paniculata leaf extract. Their analysis demonstrated that the phenolic hydroxyl and carbonyl groups within the extract functioned as both stabilizing agents and recognition sites; the injection of Pb^2+^ upset the surface electrostatic equilibrium and promoted fast particle aggregation. This probe displayed a strong response to Pb^2+^ within a linear range of 0–100 µM (LOD 12.661 µM) while displaying “multifunctional” potential for the catalytic degradation of organic dyes and bacteriostasis. To further boost detection sensitivity, Zannotti et al. [67] undertook an in-depth analysis of the AuNPs@OPE system synthesized from orange peel extract (OPE), specifically explaining the regulatory function of reaction kinetics on sensitivity. By prolonging the reaction time to enhance the coordination-driven aggregation effect, the system reached a linear range of 0.8–9.9 µM with a LOD as low as 0.05 µM, fulfilling WHO criteria for drinking water. Addressing the difficulty of multi-ion interference in complicated matrices, the researchers introduced chemometric approaches in a subsequent study on AgNPs@OPE [68]. By combining Principal Component Analysis (PCA) and Linear Discriminant Analysis (LDA), they efficiently resolved the spectrum overlap between Pb^2+^ and Cd^2+^ (accuracy 98.5%). Hladun et al. [69] built a universal colorimetric probe utilizing ascorbic acid based on the complexation mechanism between metal ions and hydroxyl groups, achieving simultaneous detection of Pb^2+^ (LOD 5.4 ppb) and Cr^6+^.

**Figure 6:**
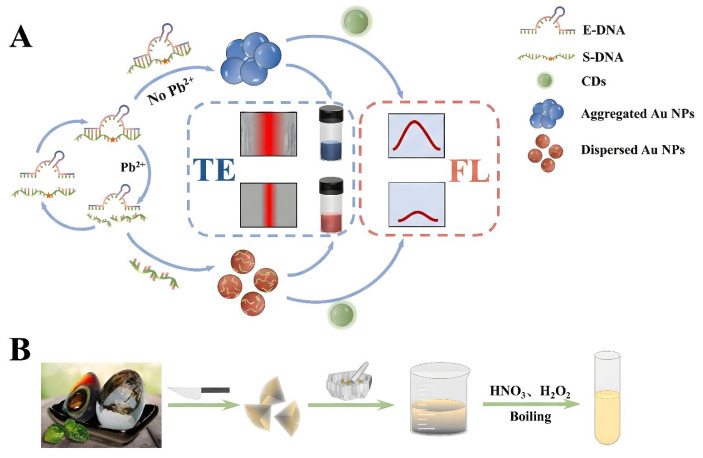
Schematic representation of the DNAzyme-mediated dual-modal sensing approach for Pb^2+^. (**A**) Schematic diagram of dual-channel detection of lead ion. (**B**) The treating process of preserved egg samples [70].

The implementation of highly specific biological enzymes signifies an additional method for enhancing quantitative precision. Yan et al. [70] deviated from the traditional “metal-ion-induced direct aggregation” model by developing a Pb^2+^ sensor utilizing DNAzyme cleavage. In this system, Pb^2+^ functions as a cofactor to activate the DNAzyme, facilitating the cleavage of the substrate strand and resulting in the disruption of single-stranded DNA modified on the AuNP surface, thus preserving the dispersed state of the AuNPs (red); conversely, in the absence of Pb^2+^, salt-induced aggregation transpires (blue). This technique adeptly transforms the alteration in dispersion state into a dual-modal signal (Figure 6A): fluorescence quenching of carbon quantum dots (CDs) by scattered AuNPs (FRET mechanism) and fluctuations in the Tyndall scattering effect induced by aggregates. This dual-channel technique enabled ultrasensitive detection of Pb^2+^, exhibiting a colorimetric linear range of 2.4 × 10^−14^ to 8.0 × 10^−10^ mol/L and a LOD as low as 0.11 pM, underscoring its significant practical utility in complicated food matrices, including preserved eggs (Figure 6B).

Despite the advancements in enzymatic cleavage strategies that have significantly enhanced sensitivity, DNAzyme-AuNP systems frequently face a limitation of sluggish dissociation kinetics in practical applications, attributable to the excessive stability of the fully matched duplex structure, which restricts the signal response rate. Liu et al. [71] introduced a “mismatch acceleration” technique to tackle this thermodynamic and kinetic problem. The incorporation of A-C mismatch base pairs into the substrate strand greatly diminished duplex stability (lower T_m_), hence significantly expediting the Pb^2+^-induced aggregation breakdown and redispersion process while preserving specificity. This approach attained a rapid response through mismatch sequence optimization within a linear range of 10–300 nM, with a detection limit of 8.6 nM.

Nkanyezi Penuel Kubheka et al. [72] developed a cost-effective quantitative method utilizing ImageJ (version 1.54p) image processing software to mitigate the reliance of traditional colorimetry on analytical instrumentation. The approach reached a limit of detection of 0.01 mg/L within a linear range of 0.1–20 mg/L by employing the color transition caused by Pb^2+^-mediated AuNP aggregation alongside smartphone RGB analysis, demonstrating performance akin to that of complicated customized probes.

Moreover, concerning the detection of the heavy metal cadmium (Cd^2+^) in cosmetics, He et al. [73] suggested a sensing approach employing unmodified AuNPs modulated by the cationic dye SYBR Green I and a particular aptamer. This process utilizes the strong attraction between Cd^2+^ and the aptamer to cause the desorption of the aptamer from the AuNP surface. As a result, the unshielded nanoparticles experience charge neutralization and significant aggregation due to electrostatic interactions with the dye, resulting in a LOD of 0.27 µM and a linear detection range of 0.2–4 µM. Munazza Arain et al. [74] advanced the approach of small-molecule functionalization by synthesizing Secnidazole-modified AgNP probes in a single step. This system utilizes the pronounced affinity of Cd^2+^ for the hydroxyl groups of surface ligands to facilitate ligand desorption and subsequent nanoparticle aggregation, attaining a low LOD of 0.021 µM within a linear range of 5–27 µM, and was effectively employed for detection in complex biological samples, including plasma.

Ghosh and Mondal [75] presented a dual-purpose spectrum quantification technique utilizing garlic extract-stabilized AgNPs modified with ascorbic acid for the development of robust platforms for multi-ion detection. The system attained a limit of quantification (LOQ) of 0.1 µM for Hg^2+^ within a linear range of 0.1–50 µM by monitoring the “Absorption Minima” in the UV region to reduce signal non-linearity. The sensor exhibited a distinct ability for colorimetric detection of Cd^2+^ through a coordination mechanism involving functional groups. This method enabled the quantitative assessment of Cd^2+^ with a limit of quantification of 5 µM and a working concentration range of 5–50 µM.

**Table 2:** Analytical efficacy of colorimetric tests for the identification of mercury (Hg^2+^) and lead (Pb^2+^) ions.

Nanostructure	Ligand	Linear Range	LOD	Method	Evaluation	Ion	Ref.
AgNPs	Citrate (Laser Ablation)	—	1.0 μM (0.2 ppm)	Physical Assistance and Post-Treatment	—	Hg^2+^	[59]
AgNPs	*Diospyros kaki* extract	0.5 nM–500 μM (0.1–100,000 ppb)	0.5 nM (0.1 ppb)	Green synthesis strategies	Wide linear range	Hg^2+^	[60]
AgNPs	CMC (*Water hyacinth*)	5–45 μM	3.14 μM	Green synthesis strategies	—	Hg^2+^	[61]
AgNPs	*Salvia tiliifolia* extract	0.1–100 μM	0.27 nM	Green synthesis strategies	Ultrasensitive	Hg^2+^	[62]
Ps-AgNPs	DNA Aptamer	2.5–100 μM (0.5–20 ppm)	2.5 μM (0.5 ppm)	Solid-Phase Support & Multimodal sensing	Sensitive & Portable	Hg^2+^	[63]
AuNPs	DNA (Probe-blocker)	0.005–1 μM	2.85 nM	Ligand Engineering & Smart Readout	Sensitive & Portable	Hg^2+^	[64]
Rib-AuNPs	Ribavirin	—	3.64 nM	Ligand Engineering	—	Hg^2+^	[65]
AuNPs	*Andrographis paniculata* extract	0–100 μM	12.661 μM	Green synthesis strategies	—	Pb^2+^	[66]
AuNPs	Orange peel extract (OPE)	0.8–9.9 μM	0.05 μM	Green synthesis strategies & Special Response Mechanisms	—	Pb^2+^	[67]
AuNPs & CDs	DNAzyme	2.4 × 10^−14^–8.0 × 10^−10^ M	0.11 pM	Ligand Engineering & Multimodal sensing	Ultrasensitive	Pb^2+^	[70]
AuNPs	DNAzyme (Mismatch)	10–300 nM	8.6 nM	Special Response Mechanisms	—	Pb^2+^	[71]
AuNPs	Unmodified	0.48–96.5 μM (0.1–20 mg/L)	48 nM (0.01 mg/L)	Smart Readout and Algorithmic Enhancement	Sensitive & Portable	Pb^2+^	[72]
AuNPs	Aptamer/SYBR Green I	0.2–4 µM	0.27 µM	Ligand Engineering	—	Cd^2+^	[73]
AgNPs	Secnidazole	5–27 µM	0.021 µM	Ligand Engineering	Ultrasensitive	Cd^2+^	[74]
AgNPs	*Allium sativum* extract/Ascorbic acid	5–50 µM	5 µM (LOQ)	Green synthesis strategies	—	Cd^2+^	[75]

### 3.2. Transition Metal Ions

This section addresses the colorimetric detection of iron (Fe), nickel (Ni), and copper (Cu) ions (Table 3). Iron ions mostly occur in divalent (Fe^2+^) and trivalent (Fe^3+^) oxidation states in biological and environmental contexts, with the choice of surface ligands being crucial for differentiating between these valences.

Contemporary approaches for colorimetric iron detection focus on attaining valence-specific recognition and improving practical applicability via ligand-to-metal charge transfer processes and gel matrix confinement. Dayanidhi et al. [76] illustrated the capability of employing saponins from Sapindus mukorossi extract as reducing agents and recognition ligands for valence-specific recognition. The research uncovered a distinct mechanism involving ligand-to-metal charge transfer (LMCT): oxygen atoms with high-energy lone pair electrons in the saponin backbone engage with Fe^2+^ or Fe^3+^ that have low-energy unoccupied 3d^0^ orbitals. This interaction produced unique spectral responses: Fe^2+^ increased the SPR band intensity and altered the solution color to black, whereas Fe^3+^ diminished the SPR band and transformed the solution to white. The probe effectively attained precise distinction between Fe^2+^ (LOD 1 µM) and Fe^3+^ (LOD 5 µM), exhibiting a linear detection range of 0–100 µM for both ions.

**Figure 7:**
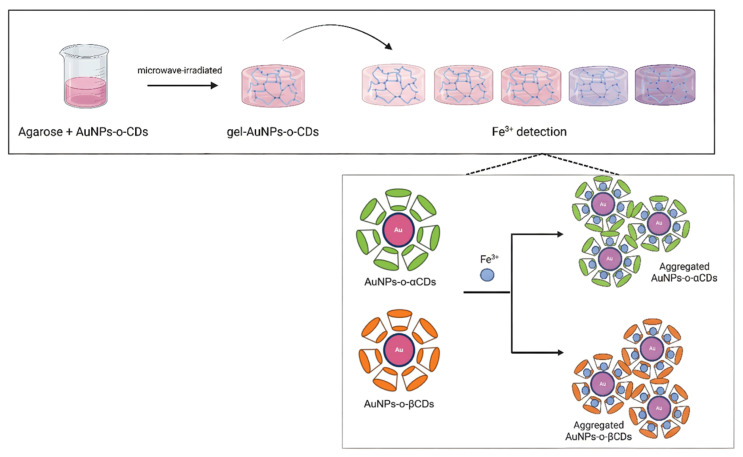
Schematic representation of the fabrication process for gel-based AuNPs sensors and the colorimetric detection technique for Fe^3+^ [77].

To further address the challenges of portability and non-specific aggregation associated with liquid-phase probes in practical applications, Andreani et al. [77] proposed a gel matrix confinement strategy (Figure 7). They synthesized AuNPs using α-cyclodextrin and β-cyclodextrin as stabilizers and integrated them into an agarose gel matrix. The cavity structure of the CDs and the gel network synergistically prevented non-specific aggregation, while the addition of Fe^3+^ induced the controlled aggregation of AuNPs, resulting in a color transition of the gel from pink to purple (Figure 7, top panel). In particular, the sensor based on β-CDs, benefiting from superior stabilizing capability and smaller particle size (~17.13 nm), demonstrated excellent performance (LOD 0.20 mg/L, linear range 2–18 mg/L).

**Figure 8:**
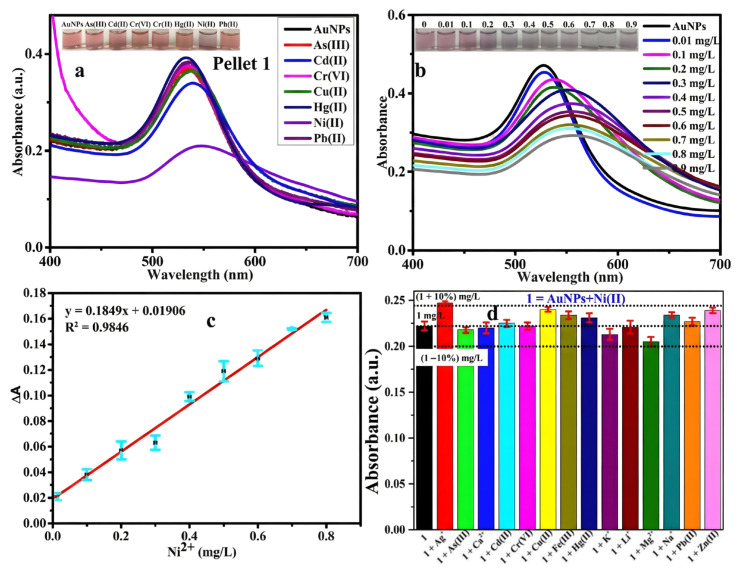
Colorimetric sensing efficacy of the bio-synthesized AuNPs for Ni^2+^. (**a**) UV-Visible spectra of pellet 1 interacted with different metal ions (5 mg/L) (Inset images show the color change of pellet 1). (**b**) UV–Vis spectra of AuNPs in response to varying concentrations of Ni(II). The inset image illustrates a gradual shift in color, transitioning from pink to blue as AuNPs react with Ni(II) at concentrations ranging from 0.001 to 1 mg/L. (**c**) Associated standard graph plotted against Ni(II) concentration. (**d**) The specificity of AuNPs for Ni(II) at a concentration of 1 mg/L in the presence of 1.5 times concentrations of various other metal ions [78].

The principal obstacle in the colorimetric detection of nickel ions (Ni^2+^) is the efficient removal of interference from concurrent divalent ions. Enhancing surface ligand chemistry alongside digital image colorimetry (DIC) can markedly improve the specificity and precision of Ni^2+^ detection. Patra et al. [78] utilized green tea extract for the eco-friendly synthesis of AuNPs, employing rich polyphenols, including gallocatechin and epicatechin gallate, as functional capping agents. The hydroxyl (–OH) and carbonyl (C=O) functional groups on these biomolecules provide several coordination sites for Ni^2+^, facilitating electrostatic attraction and coordination binding between the positively charged Ni^2+^ and the negatively charged AuNP surface. This interaction caused a red shift in LSPR absorption peak from 528 nm to 556 nm, along with a color change in the solution from pink to purple (Figure 8a,b). The probe demonstrated exceptional anti-interference capability regarding Ni^2+^ (Figure 8d), exhibiting a robust linear response within the range of 0.001–1 mg/L (Figure 8c) and achieving a LOD as low as 0.001 mg/L, surpassing World Health Organization (WHO) standards, thereby validating the effectiveness of natural polyphenol ligands in the specific recognition of Ni^2+^.

Nubatonis et al. [79] devised a detection strategy utilizing chemical ligand-functionalized AgNPs integrated with smartphone-based DIC analysis for the specific detection of Ni^2+^. Unlike intricate biological extracts, they employed mercaptosuccinic acid (MSA) and ethylenediaminetetraacetic acid (EDTA) to alter AgNPs. They facilitated AgNP aggregation by utilizing the particular binding sites of carboxyl, amino, and thiol groups through robust Ni^2+^-ligand coordination. This induced a color transition in the solution from yellow to blue and a notable shift in the LSPR peak from 402 nm to 620 nm. This method attained a LOD of 3.57 µM and a linear detection range of 10–300 µM for Ni^2+^ by transforming image RGB values into Euclidean distances for quantitative analysis. This research illustrates that the integration of meticulously designed chemical ligands with sophisticated imaging algorithms significantly improves the accuracy of on-site detection of Ni^2+^.

Wu et al. [80] devised a green, ligand-free gold nanoparticle probe for the detection of copper ions (Cu^2+^). Polysaccharides and proteins from the alga Padina australis chelated with Cu^2+^ through their –NH_2_ and –OH groups, causing the aggregation of AuNPs and leading to a notable red shift (from 520 nm to 630 nm). This technique is straightforward and effective, exhibiting commendable linearity for the quantitative measurement of Cu^2+^ within the range of 20–60 µM, with a LOD of 0.43 µM. Likewise, Aqillah et al. [81] employed the traditional citrate reduction technique to synthesize AuNPs, utilizing a detection methodology predicated on Cu^2+^-induced aggregation through citrate displacement or bridging. Their research concentrated on enhancing a smartphone-based digital image colorimetry (DIC) readout system, attaining accuracy that surpasses conventional UV-Vis spectroscopy by analyzing the blue channel.

Nguyen and colleagues [82] proposed an approach for in situ hydrogel synthesis. They utilized L-cysteine to decrease HAuCl_4_ within an agar hydrogel matrix. In contrast to liquid-phase environments, the agar hydrogel functioned as a dynamic regulator of molecular diffusion, offering structural support while simultaneously improving dispersion stability by limiting the Brownian motion of the AuNPs. Cu^2+^ acted as a cross-linker, interacting with the functional groups (–SH, –COOH, –NH_2_) of L-cysteine to promote aggregation. This method attained a LOD of 0.65 µM and a linear range of 10 to 70 µM. This “matrix-assisted” approach substantially mitigated the problem of spontaneous aggregation frequently encountered in conventional colloidal probes (solidification of liquid-phase probes).

**Figure 9:**
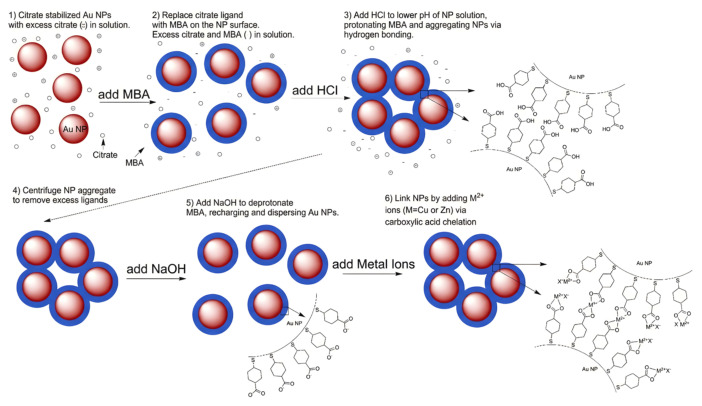
Schematic illustration of the ligand exchange and pH-selective precipitation (PSP) purification method [83].

Colford and Dhirani [83] presented a novel “pH-selective precipitation (PSP)” purification process to address the influence of surface ligand purity on sensitivity. They indicated that remaining excess capping agents from conventional production can competitively bind metal ions, thus reducing sensor sensitivity. The researchers generated mercaptobenzoic acid (MBA)-functionalized AuNPs and utilized the PSP technique, adjusting pH to facilitate reversible aggregation and redispersion for the removal of excess citrate and MBA (Figure 9). The purified probes (MBA-AuNPs) demonstrated remarkable sensitivity to Cu^2+^, as the particular chelation between Cu^2+^ and carboxyl groups prompted the formation of aggregates (Figure 9, step 6). This approach boosted the LOD by 100-fold (achieving 10^−5^ M) compared to unpurified probes, and the direct coordination of Cu^2+^ to the MBA monolayer was verified using X-ray photoelectron spectroscopy (XPS) and SERS. This discovery highlights the essential importance of ligand layer purification in the development of ultrasensitive colorimetric sensors.

**Table 3:** Analytical efficacy of colorimetric tests for the identification of iron (Fe), nickel (Ni^2+^), and copper (Cu^2+^) ions.

Nanostructure	Ligand	Linear Range	LOD	Method	Evaluation	Ion	Ref.
Ag/Au NPs	Saponins	0–100 μM	1 μM	Green synthesis strategies & Special Response Mechanisms	—	Fe^2+^/^3+^	[76]
AuNPs	Cyclodextrin (Gel matrix)	35.8–322 μM (2–18 mg/L)	3.6 μM (0.20 mg/L)	Solid-Phase Support and Phase Transition	—	Fe^3+^	[77]
AuNPs	Green tea extract	17 nM–17 μM (0.001–1 mg/L)	17 nM (0.001 mg/L)	Green synthesis & Smart Readout		Ni^2+^	[78]
AgNPs	MSA & EDTA	10–300 μM	3.57 μM	Ligand Engineering & Smart Readout	Sensitive & Portable	Ni^2+^	[79]
AuNPs	*Padina australis* polysaccharides	20–60 μM	0.43 μM	Green synthesis strategies	—	Cu^2+^	[80]
AuNPs	L-Cysteine (Hydrogel)	10–70 μM	0.65 μM	Solid-Phase Support and Phase Transition	—	Cu^2+^	[82]
AuNPs	MBA (Purified)	—	10 μM	Physical Assistance and Post-Treatment	—	Cu^2+^	[83]

### 3.3. Other Metal Ions

This section addresses the colorimetric detection of aluminum (Al^3+^) and various metal ions. A major technique for attaining selective recognition of Al^3+^ includes altering noble metal nanoparticles with natural or synthesized small molecules rich in oxygen and nitrogen donors, which induce aggregation via metal-ligand interactions. Joshi et al. [84], Rastogi et al. [85], and Ghodake et al. [86] utilized indole-2-carboxylic acid, ascorbic acid, and gallic acid as functional ligands, respectively, to construct extremely sensitive silver/gold nanoprobes. The limits of detection (LOD) were 0.01 ppm, 12.5 ppb, and 1.48 µM, respectively, with refs. [84,85] reporting linear ranges of 0.5–10 ppm and 100–350 ppb, respectively. Building on this foundation, important innovations have further increased detection functionalities: Bezuneh et al. [87] introduced a competitive coordination mechanism using tannic acid-functionalized AgNPs, achieving dual detection of Al^3+^ (LOD 0.2 µM, linear range 2–25 µM) and F^−^; meanwhile, Montenegro et al. [88] modified AgNPs with carbon dots and combined them with chemometric modeling (MCR-ALS) to quantitatively resolve the binding kinetics of Al^3+^ (LOD 3.5 µM).

Taheri and Khayatian [89] developed an economical microfluidic chip utilizing poly (methyl methacrylate) (PMMA) and cotton thread for on-site portable detection. This research utilized ammonium pyrrolidine-1-carbodithioate (APDC) to alter AgNPs, leveraging the particular interaction between the nitrogen and sulfur atoms in APDC and the metal ions to promote aggregation, thus facilitating concurrent colorimetric detection of Al^3+^ and Cr^3+^. The introduction of NaF as a masking agent effectively mitigated interference from concurrent ions. The limits of detection for Al^3+^ and Cr^3+^ were 3.55 nM and 10.66 nM, respectively, with linear detection ranges of 0.01–250 µM and 0.1–220 µM, respectively, confirming the applicability of microfluidic technology in multi-component environmental studies.

In relation to the essential technological component tellurium (Te^4+^), Kim et al. [90] employed natural alginate as a bifunctional agent for the environmentally friendly synthesis of AgNPs. Based on the chelation of Te^4+^ with surface carboxyl/hydroxyl groups inducing ligand dissociation and particle aggregation, this method effectively shields interference from congeneric elements (As, Se), achieving a LOD of 22 nM and a linear response range of 31.3–407.5 nM, validating its applicability in environmental water analysis. For the detection of the rare earth element La^3+^, Hussain et al. [91] developed a smartphone-based paper sensing platform utilizing resorcinarene-modified AgNPs. Relying on an aggregation mechanism generated by the particular coordination of La^3+^ with macrocyclic oxygen atoms, colorimetric identification from yellow to gray was obtained. This approach revealed a good linear response to La^3+^ in the range of 0.05–100 μM, with a LOD of 15 nM.

The colorimetric detection of alkali metal ions fundamentally depends on electric double-layer compression and the alteration of colloidal stability. Hsiao et al. [92] examined the influence of AuNP surface potential on the sensitivity of Na^+^ detection. The research indicated that the Zeta potential of AuNPs rose from −87.7 mV to −58.2 mV following treatment with ascorbic acid, resulting in a metastable condition. The addition of Na^+^ diminished the activation energy for aggregation, measured at 22.5 kJ·mol^−1^, in accordance with the ionic strength-induced aggregation outlined by DLVO theory. This probe accomplished visual detection of Na^+^ (LOD around 30 mM) via straightforward electrostatic interactions, and a second-order polynomial regression model was developed for quantitative analysis, offering a cost-effective approach for high-concentration salt detection. Berasarte et al. [93] established a universal electrolyte detection platform for Na^+^, K^+^, Ca^2+^, and Mg^2+^ via lysine-assisted AgNP aggregation. Lysine served as a crucial inducer, facilitating AgNP aggregation and color alteration in the presence of electrolytes. This study’s novelty resides in the thorough integration of digital image analysis (DIA) technologies, utilizing principal component analysis (PCA) for particle optimization and partial least squares (PLS) models to mitigate K^+^ interference on Na^+^, ensuring a relative inaccuracy of no more than 13%. This technique, which integrates multivariate calibration with smartphone imaging, signifies an advancement of colorimetric sensors towards enhanced portability and intelligence.

Patel et al. [94] introduced a morphology-regulated “multicolor” sensing method, employing bovine serum albumin (BSA) as a bifunctional reagent to produce silver nanostructures with varying aspect ratios and starting hues (yellow, orange, green, blue). This study demonstrated that the ion response of the probes exhibits considerable “shape dependence”: heavy metal ions (Cr^3+^, Hg^2+^) predominantly induce characteristic red-shifted aggregation of spherical particles (yellow/orange) through coordination with surface amino or carboxyl groups of BSA; conversely, the alkali metal K^+^, which is challenging to complex, specifically destabilizes plate-like nanostructures (green/blue) and induces a blue shift (hypsochromic shift) in the absorption spectra, thereby facilitating visual differentiation of multiple ions in complex matrices based on distinct spectral evolution patterns.

## 4. SERS Sensors

The aggregation of nanoparticles causes significant alterations in the LSPR spectrum and concurrently activates robust electromagnetic coupling effects at the microscopic level. SERS detection principally utilizes the high-density electromagnetic “hotspots” created within the interparticle gaps of aggregates to exponentially enhance the signals of molecules situated in these areas. This technology allows for the dynamic modulation of local enhancement factors by changing the assembly or disassembly state of nanoparticles through target ions, facilitating quantitative study of targets based on “Signal-on” or “Signal-off” Raman mechanisms.

### 4.1. Heavy Metal Ions

This section evaluates the analytical performance of the SERS assays for heavy metal ion detection. The diagnostic efficacy and detection limits for lead Pb^2+^ and mercury Hg^2+^ ions are summarized in Table 4. Subsequently, the analytical metrics for the identification of cadmium Cd^2+^ and chromium Cr^3+^ ions are presented in Table 5.

Lead ions (Pb^2+^) exhibit considerable biological toxicity and can engage in robust interactions with sulfur- and nitrogen-containing groups in animals. Their accumulation in environmental and biological systems presents significant hazards to the nervous system. The coordination interaction between Pb^2+^ and surface-modified ligands to promote nanoparticle aggregation is a crucial method for manufacturing SERS probes. Frost et al. [95] devised an efficient SERS sensing device utilizing citrate-functionalized AuNPs (~6 nm). This study utilized citrate molecules as dual-functional agents serving as colloidal stabilizers and identification probes, yielding a highly stable colloidal solution with a surface charge of −44.3 mV. Upon the introduction of Pb^2+^, it exhibits robust coordination with the carboxyl (COO^−^) and hydroxyl (OH) groups on the citrate surface, resulting in the aggregation of AuNPs. Under 514 nm laser excitation, this aggregation process creates efficient SERS hotspots, resulting in a significant reduction in the citrate v(O-H) vibrational peak strength (about 3200 cm^−1^) as Pb^2+^ concentration increases. The approach demonstrates a linear response range of 50–1000 ng/L and a LOD of 25 ng/L, and it has been effectively utilized for fast detection in aqueous environmental systems.

**Figure 10:**
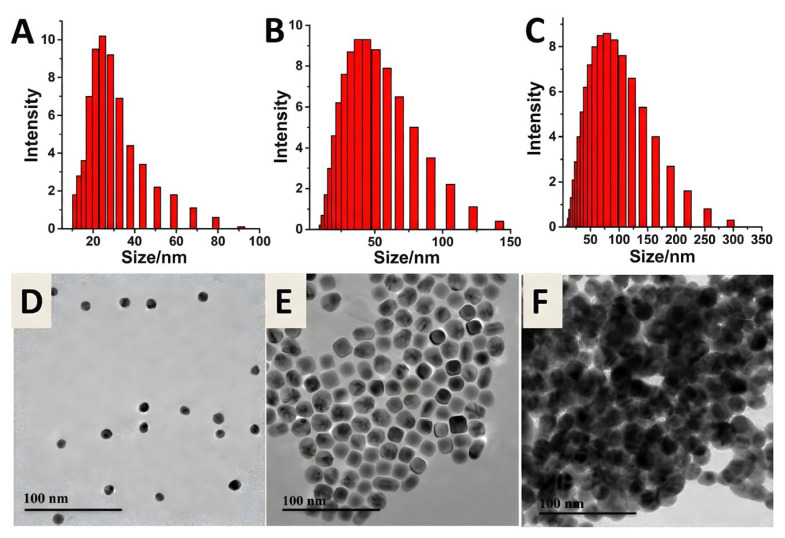
Mean average hydrodynamic diameters of (**A**) Au@Ag NPs, (**B**) Au@Ag NPs probes and (**C**) Au@Ag NPs probes aggregations in the presence of Pb^2+^. Transmission electron microscopy (TEM) of (**D**) Au@Ag NPs and (**E**) Au@Ag NPs probes and (**F**) Au@Ag NPs probes aggregations in the presence of Pb^2+^ [96].

Xu et al. [96] developed a sensing platform utilizing L-cysteine (L-cys) functionalized Au@Ag core–shell nanoparticles (Au@Ag NPs). In contrast to monometallic AuNPs, the Au@Ag NPs merge the stability of the gold core with the enhanced dielectric characteristics of the silver shell, exhibiting superior localized electromagnetic field enhancement capabilities. In this system, Pb^2+^ selectively chelates with the carboxyl and amino groups of L-cysteine, causing the Au@Ag nanoparticles to shift from a dispersed to an aggregated form, resulting in a substantial amplification of the Raman signal of the reporter molecule, 4-aminothiophenol (4-ATP), under 532.8 nm laser excitation. The assembly process is clearly validated by TEM and DLS data, as illustrated in Figure 10. The creation of the Au@Ag core–shell structure results in a hydrodynamic diameter of approximately 25.5 nm; subsequent injection of Pb^2+^ causes the nanoparticles to shift from a monodisperse state to separate agglomerates (Figure 10F), resulting in a substantial rise in average particle size. These nanoscale aggregates offer numerous electromagnetic hotspots for SERS signal amplification. This method efficiently mitigates interference from ions such as Hg^2+^ by employing potassium thiocyanate (KSCN) as a masking agent, resulting in a low LOD of 1 pM and a logarithmic linear standard curve spanning from 5 pM to 10 nM. Furthermore, the probe exhibited satisfactory stability (>80% activity after 15 days), suggesting its potential for practical applications.

Anisotropic nanomaterials can produce more potent electromagnetic field enhancement effects at their tips owing to their geometric configurations. Liu et al. [97] engineered gold-core silver-shell nanorods (Au@Ag NRs) co-modified with GSH and 4-MBA. GSH is tethered through Ag-S interactions, and its accessible carboxyl groups, in conjunction with 4-MBA, function as binding sites for Pb^2+^. The introduction of Pb^2+^ promotes the self-assembly and aggregation of the nanorods, resulting in the formation of high-density hotspots in the interparticle spaces, which markedly amplifies the distinctive peak of 4-MBA at 1072 cm^−1^ under 785 nm excitation. This probe has a LOD of 0.021 µg/L and a linear range of 0.5 to 1000 µg/L. Notably, the signal intensity remained >87% over three weeks, proving its high stability. Combined with the reported low cost, this method shows great potential for portable, large-scale on-site food safety screening, as validated in tea powder and sticky rice flour.

Strategies for DNAzyme functionalization employ specialized catalytic cleavage reactions activated by metal ions to accurately control the surface properties and aggregation behavior of nanoparticles, hence enhancing detection selectivity considerably. Liu et al. [98] introduced a SERRS sensing system that integrates DNAzyme with ~20 nm Au/Ag NPs under 633 nm excitation. This approach utilizes the catalytic activity of Pb^2+^ to cleave the substrate DNA strand, in contrast to the previously reported small-molecule ligand-induced aggregation. In the presence of Pb^2+^, the cleaved single-stranded DNA adheres to the nanoparticle surface, resulting in the development of “loose aggregates” of Au/Ag NPs with elevated SERRS activity, thereby markedly amplifying the signal of Rhodamine 6G (RhG)**.** In the absence of Pb^2+^, the undamaged double-stranded DNA fails to offer adequate protection, resulting in tight aggregation of the system and yielding only a faint background signal. This technique adeptly utilizes the specificity of DNAzyme, attaining a limit of detection of 7 × 10^−9^ M and a linear detection range of 5.0 × 10^−8^ to 6.0 × 10^−7^ mol/L, while exhibiting remarkable anti-interference properties.

Wang et al. [99] were pioneers in developing a sensing system for ~35 nm AgNPs using T-rich aptamers under 633 nm excitation. Utilizing the notion that Hg^2+^ prompts the aptamer to fold into a T-Hg^2+^-T configuration, thereby diminishing its protective function, the method incorporated cationic spermine to facilitate nanoparticle aggregation. This accomplished a “Signal-on” SERS detection of the surface-labeled chemical TAMRA (LOD 5 nM). While physical aggregation procedures are efficient, chemical mechanisms involving amalgam production between mercury ions and silver are more commonly utilized in SERS detection with silver-based nanomaterials. The research conducted by Hassan et al. [100] frequently illustrates this idea of non-aggregation. This work employed Au@Ag nanoparticles as substrates; in the presence of Hg^2+^, a distinct reaction transpired with the silver shell to produce Ag-Hg amalgam. This chemical reaction modified the surface plasmon resonance characteristics of the nanoparticles and resulted in the desorption or displacement of the surface-adsorbed signal molecule (R6G), which macroscopically appeared as a substantial reduction in the SERS signal with rising Hg^2+^ concentration (Signal-off).

Dasary et al. [101] devised an ultrasensitive detection method for cadmium ions (Cd^2+^) utilizing multi-ligand synergistic effects. They employed Alizarin as the Raman reporter molecule and incorporated 3-mercaptopropionic acid (MPA) and 2,6-pyridinedicarboxylic acid (PDCA) to alter 13 nm AuNPs. Under conditions of pH 8.5, Cd^2+^ established a stable hexadentate coordination complex with the surface ligands, resulting in particle aggregation and eliciting substantial electromagnetic field coupling effects, which amplified the distinctive peak of Alizarin at 1335 cm^−1^ by about 10^7^-fold under 670 nm excitation. This approach is distinguished by its exceptional sensitivity (LOD as low as 10 ppt) and reversibility under EDTA regulation, demonstrating that the aggregation process arises from ion-template chelation. Notably, the functionalized AuNPs remained stable for over 10 days in buffer solutions, indicating great potential for prototype scale-up. Du and Jing [102] presented a “one-pot” technique to streamline the synthesis process, employing dopamine (DA) as both a reducing and capping agent to fabricate functionalized AuNPs, thereby developing a sensor based on a “Signal-on” mechanism. The quinone moieties in the oxidation products of dopamine demonstrate selective recognition for Cd^2+^ ions. The investigation of two-dimensional correlation spectroscopy (2D-COS) verified that the robust chelation between quinone groups and Cd^2+^ prompted swift aggregation of AuNPs, leading to a substantial amplification of the Raman signal at 1618 cm^−1^ **under** 785 nm excitation. This probe attained a detection limit of 10^−8^ M, exhibiting a linear range from 10^−4^ M to 10^−8^ M, and showcased remarkable anti-interference efficacy in intricate matrices. In contrast to the direct cross-linking technique, Guo et al. [103] employed the notion of “ligand competitive displacement” to develop an R6G/GSH/AuNPs (~40 nm) sensing platform under 632.8 nm excitation. This technique utilized GSH as a stabilizing agent. The stability of the [Cd(SG)_4_] complex, produced by Cd-S and Cd-N bonds with a binding energy of 208.5 kJ/mol, is considerably greater than that of the Au-S bond, resulting in the desorption of GSH from the surface of AuNPs in the presence of Cd^2+^. The AuNPs, having shed their protective coating, aggregated due to the presence of electrolytes, hence activating the SERS signal of Rhodamine 6G (R6G). This technique successfully circumvented non-specific adsorption problems, attaining a limit of detection of 10 ppb for Cd^2+^ within a linear range of 0.5 ppm to 20 ppm. The method exhibited good reproducibility (RSD 6.3%) and is verified as a cost-effective strategy for on-site monitoring using portable Raman systems.

In the realm of SERS detection of Cr^3+^, Ye et al. [104] developed a Tween 20-stabilized citrate-capped AuNPs system. By employing the targeted chelation of Cr^3+^ with surface citrate to mitigate steric hindrance and promote aggregation, they markedly amplified the signal of the reporter molecule (2-ATP), attaining highly selective detection within a linear range of 50–200 nM (LOD 50 nM). Ly and Joo [105] employed EDTA-modified AgNPs to demonstrate that the conformational alteration caused by the coordination of Cr^3+^ with EDTA might initiate nanoparticle aggregation. This procedure markedly amplified the metal-ligand (Cr-N) vibrational signal at 563 cm^−1^, attaining a detection threshold of 0.5 µM in seawater matrices. Cheng et al. [106] developed Au-core/Ag-shell composite nanoprobes (17.5 nm Au core/4.7 nm Ag shell) for the quantitative assessment of Cr^3+^, utilizing 4-MBA as the signaling molecule and DL-mercaptosuccinic acid (DL-MSA) as the recognition component. The precise chelation of Cr^3+^ with the terminal carboxyl groups of DL-MSA facilitated the cross-linking and aggregation of the nanoprobes, resulting in a linear increase in the signal of 4-MBA at 1585 cm^−1^ with concentration. This sensor attained an exceptionally low detection limit of 3 × 10^−10^ M and demonstrated favorable biocompatibility.

**Table 4:** Analytical efficacy of SERS tests for the identification of lead (Pb^2+^) and mercury (Hg^2+^) ions.

Nanostructure	Ligand	Linear Range	LOD	Method	Evaluation	Ion	Ref.
AuNPs	Citrate	0.24–4.8 nM (50–1000 ng/L)	0.12 nM (25 ng/L)	Molecular Probe Engineering	—	Pb^2+^	[95]
Au@Ag NPs	L-cysteine & 4-ATP	5 pM–10 nM	1 pM	Plasmonic nanohybrid design	Ultrasensitive	Pb^2+^	[96]
Au@Ag NRs	GSH & 4-MBA	2.4 nM–4.8 μM (0.5–1000 µg/L)	0.1 nM (0.021 µg/L)	Plasmonic nanohybrid design	Wide linear range	Pb^2+^	[97]
Au/AgNPs	DNAzyme	5.0 × 10^−8^–6.0 × 10^−7^ M	7 nM	Special Response Mechanisms	—	Pb^2+^	[98]
AgNPs	Aptamer/Spermine	—	5 nM	Molecular Probe Engineering	—	Hg^2+^	[99]
AgNPs	L-cysteine	—	Cu: 10 pM Hg: 1 pM	Molecular Probe Engineering	Ultrasensitive	Cu^2+^, Hg^2+^	[107]

**Table 5:** Analytical efficacy of SERS tests for the identification of cadmium (Cd^2+^) and chromium (Cr^3+^) ions.

Nanostructure	Ligand	Linear Range	LOD	Method	Evaluation	Ion	Ref.
AuNPs	Alizarin/MPA/PDCA	—	89 pM (10 ppt)	Molecular Probe Engineering	Ultrasensitive	Cd^2+^	[101]
AuNPs	Dopamine (DA)	10^−4^–10^−8^ M	10 nM	Molecular Probe Engineering	Wide linear range	Cd^2+^	[102]
AuNPs	R6G/GSH	4.45–178 μM (0.5–20 ppm)	89 nM (10 ppb)	Special Response Mechanisms	—	Cd^2+^	[103]
AuNPs	Tween 20/Citrate	50–200 nM	50 nM	Molecular Probe Engineering	—	Cr^3+^	[104]
AgNPs	EDTA	—	0.5 µM	Molecular Probe Engineering	—	Cr^3+^	[105]
Au-core/Ag-shell	4-MBA/DL-MSA	—	0.3 nM	Plasmonic nanohybrid design	Ultrasensitive	Cr^3+^	[106]

### 4.2. Transition Metal and Other Metal Ions

This section evaluates the application of SERS for the identification of copper Cu^2+^ and iron (Fe) ions. The analytical efficacy and performance metrics of these tests are presented in Table 6.

Copper ions (Cu^2+^), being redox-active metals, can function as coordination centers to promote aggregation or modify probe surface characteristics via catalytic activity. Using 20 nm AgNPs under 633 nm excitation, Li et al. [107] utilized L-cysteine-modified silver nanoparticles at an early stage. Utilizing the mechanism in which Cu^2+^ or Hg^2+^ forms insoluble inner complexes with surface ligands to promote aggregation, they successfully mitigated ionic interference by including SCN^−^ as a masking agent, resulting in high-sensitivity SERS detection of Cu^2+^ (LOD 10 pM) and Hg^2+^ (LOD 1 pM)**.** Unlike the previously stated methodologies employing exogenous probes, Ly et al. [108] introduced an innovative mechanism centered on the redox dissociation of glycine (GLY) on 40 nm AuNPs, aiming to provide a simple and inexpensive discrimination method. Cu^2+^ was found to significantly stimulate the aggregation of AuNPs and facilitate the conversion of GLY into cyano (CN) species on positively charged surfaces, resulting in a pronounced distinctive peak in the Raman silent area (~2108 cm^−1^). This technique exhibited a detection limit (LOD) of 500 nM and a linear range of 0 to 10 µM in the study of real river water and HeLa cell imaging. Xu et al. [109] investigated the dual role of polyvinylpyrrolidone (PVP) as a stabilizer and a recognition element in an ~8 nm AgNPs system excited at 532 nm. They verified that Cu^2+^ functions as a cross-linker to facilitate aggregation and developed a ratiometric detection method utilizing the intensity ratio of PVP’s intrinsic Raman peaks (I_845_/I_899_), attaining a linear range of 0.01–2 µM and a detection limit of 3 nM. To tackle the intricate background interference in biological matrices, Wang et al. [110] developed a probe co-modified with L-cysteine and 4-mercaptobenzonitrile. In a similar manner, they employed copper ion-induced aggregation to activate the signal in the Raman quiet region (2220 cm^−1^), thereby accomplishing interference-free intracellular detection (LOD 0.055 µM, linear range 1 µM to 10 mM). Crucially addressing safety and stability concerns for biological applications, they demonstrated the probe’s low cytotoxicity (>90% cell viability) and excellent long-term stability (up to 15 days).

**Figure 11:**
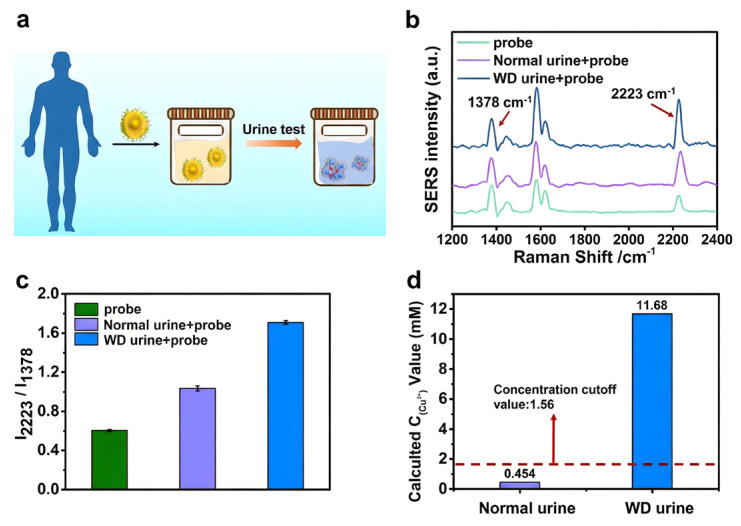
Practical implementation of the ratiometric SERS probe for the diagnosis of Wilson’s disease (WD) (**a**) Illustration of in vitro detection of urinary Cu^2+^ of a WD patient using AuNNP-NAT@MBN/PNIPAM. (**b**) SERS spectra of 0.866 nM AuNNP-NAT@MBN/PNIPAM (probe), urine of a WD patient incubated with 0.866 nM AuNNP-NAT@MBN/PNIPAM at 37 °C for 4 h (WD urine + probe), and urine of a normal person incubated with 0.866 nM AuN-NAT@MBN/PNIPAM at 37 °C for 4 h (normal urine + probe). (**c**) SERS signal intensity ratio of I2223/I1378 obtained from (**b**). Error bars represent standard deviation (n = 3). (**d**) Calculated concentration of Cu^2+^ in the urine samples of a WD patient (WD urine) and normal person (normal urine), respectively. The cutoff value of urinary Cu^2+^ concentration to differentiate the WD patient and the normal person was 1.56 mM [111].

Feng et al. [111] developed a smart probe utilizing ~52 nm PNIPAM-functionalized gold nanogap particles (AuNNPs) under 633 nm excitation for clinical in vitro diagnostics. This technique employed a Cu^2+^ coordination-induced particle aggregation mechanism to attain accurate SERS measurement of Cu^2+^ (LOD 57.4 µM, linear range 0–18 mM) by analyzing the signal ratio of the surface reporter molecule (MBN) to the internal standard molecule (NAT) (I_2223_/I_1378_). The ratiometric technique illustrated in Figure 11 exhibited significant practical use in the screening of Wilson’s disease (WD). The probe successfully mitigated urine matrix interference by leveraging the self-calibration effect of the internal standard. Detection results indicated that Cu^2+^ concentrations in urine samples from Wilson’s disease patients (~11.68 mM) were markedly elevated compared to those in the healthy control group (~0.454 mM) and the clinical diagnostic threshold (1.56 mM), hence affirming the efficacy and excellent biocompatibility (verified in mice) of this approach for early illness identification.

To tackle the problem of intricate matrix interference in real water bodies, Hsieh and Huang [112] developed magnetic functional materials to create Fe_3_O_4_@SiO_2_-Ag-4MBA core–shell nanoprobes (excited at 532 nm). This technique integrated the twin benefits of physical magnetic aggregation and chemically induced aggregation: an external magnetic field initially concentrated and separated the probes, subsequently followed by the close aggregation of particles facilitated by the bidentate coordination between Cu^2+^ and MBA. This design not only successfully mitigated matrix effects through magnetic separation but also markedly improved sensitivity through dual aggregation (LOD 0.421 ppm, linear range 0.5–20 ppm). It demonstrated enhanced anti-interference properties, especially against Fe^2+^ and Zn^2+^, offering an effective solution for the swift on-site analysis of intricate environmental samples using portable Raman spectrometers.

The catalytic activity or redox characteristics of Cu^2+^ facilitate the development of response systems predicated on “chemical shearing” or “redispersion” mechanisms. Li et al. [113] introduced a “label-free” biomacromolecule shearing technique via a Fenton-like reaction using ~10 nm AuNPs under 785 nm excitation. This approach utilizes Cu^2+^ to catalyze ascorbic acid, producing highly oxidative hydroxyl radicals (•OH) that oxidatively split the protective layer of biomacromolecules (such as HSA, BSA, or DNA) surrounding the AuNPs, in contrast to standard coordination cross-linking. The deprotected AuNPs aggregate in a saline solution, activating the SERS signal. This “Signal-on” mechanism prevents the synthesis of intricate ligands. Benefiting from the stable nanosol substrate, this low-cost method showed high reproducibility (RSD < 2.0%). The results demonstrated that SERS had a robust linear response within the range of 0.025 to 25 μmol/L, with a detection limit of 0.008 μmol/L.

Utilizing the strong attraction of Fe^3+^ for oxygen atoms, natural extracts abundant in oxygen-containing functional groups can facilitate precise detection. Guo et al. [114] employed the exceptional affinity of iron ions for oxygen atoms to develop a 632.8 nm-excited silver nanoparticle (AgNPs) system functionalized with environmentally benign phytic acid (IP6) and modified with Rhodamine 6G (R6G). IP6, abundant in phosphate groups, establishes persistent Fe-O chelation complexes with Fe^3+^, resulting in the reduction in interparticle distances to around 2.36 nm, thereby creating high-density SERS “hotspots”. This probe exhibited excellent long-term stability (>3 months). This approach, utilizing R6G as the signal reporter, attained a LOD of 0.28 ppm and a linear range of 11.2–39.2 ppm. Moreover, the negatively charged IP6 layer efficiently protected against interference from ambient ions.

Xu et al. [115] developed a very sensitive and inexpensive label-free analytical method for Fe^2+^ detection by leveraging the synergistic effects of coordination chemistry and surface-enhanced resonance Raman scattering (SERRS) using ~30 nm AgNPs (532 nm excitation)**.** They chose 2,2′-bipyridine (Bpy) as the probe chemical. The introduction of Fe^2+^ prompts a conformational shift of Bpy from trans to cis, hence resulting in the creation of a stable [Fe(Bpy)_3_]^2+^ complex. This compound efficiently promotes AgNP aggregation to create electromagnetic hotspots and features an absorption peak at 522 nm, which closely aligns with the excitation light source at 532 nm, resulting in robust resonance Raman enhancement signals. Utilizing this dual enhancement process, the approach attained a broad linear range of 10^−11^ to 10^−7^ M and an exceptionally low detection limit of 5.73 pM. Moreover, leveraging the particular coordination configuration, the probe demonstrated exceptional anti-interference capability; the sensor preserved a distinct spectral response to Fe^2+^ even in the presence of various competing metal ions, affirming its applicability in complex environments.

**Table 6:** Analytical efficacy of SERS tests for the identification of copper (Cu^2+^) and iron (Fe) ions.

Nanostructure	Ligand	Linear Range	LOD	Method	Evaluation	Ion	Ref.
AuNPs	Glycine (GLY)	0–10 µM	500 nM	Special Response Mechanisms	—	Cu^2+^	[108]
AgNPs	PVP	0.01–2 µM	3 nM	Molecular Probe Engineering	Self-calibrated	Cu^2+^	[109]
AgNPs	L-Cys & 4-MBN	1 µM–10 mM	0.055 µM	Molecular Probe Engineering	—	Cu^2+^	[110]
AuNNPs	PNIPAM/MBN	0–18 mM	57.4 µM	Molecular Probe Engineering	Self-calibrated	Cu^2+^	[111]
Fe_3_O_4_@SiO_2_-Ag	4-MBA	7.9–315 μM (0.5–20 ppm)	6.6 μM (0.421 ppm)	Plasmonic nanohybrid design	—	Cu^2+^	[112]
AuNPs	Ascorbic acid/BSA	0.025–25 µM	8 nM	Special Response Mechanisms	—	Cu^2+^	[113]
AgNPs	Phytic acid (IP6)	200–700 μM (11.2–39.2 ppm)	5 μM (0.28 ppm)	Green Synthesis Strategies	—	Fe^3+^	[114]
AgNPs	2,2′-bipyridine (Bpy)	10^−11^–10^−7^ M	5.73 pM	Molecular Probe Engineering		Fe^2+^	[115]

**Figure 12:**
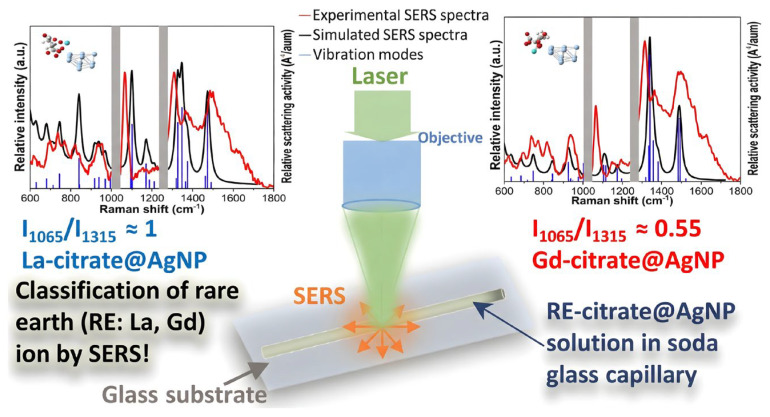
SERS differentiation of La^3+^ and Gd^3+^ with citrate-capped AgNPs. The spectra display unique intensity ratios (I_1065_/I_1315_) resulting from spin-state variations (4f^0^ vs. 4f^7^), validated by DFT computations [118].

Functionalized AgNPs have been thoroughly investigated for SERS-based detection of diverse metal and metalloid ions. Li et al. [116] were pioneers in developing ~65 nm AgNP probes (632.8 nm excitation) co-modified with GSH and 4-mercaptopyridine (4-MPY) for the detection of the very hazardous metalloid arsenic (As^3+^). By exploiting the unique affinity of As^3+^ for the oxygen-rich moieties in GSH (As-O linkages) to promote the aggregation of silver nanoparticles, they amplified the SERS signal of the surface beacon 4-MPY, attaining high-sensitivity detection of trace As^3+^ in water (LOD 0.76 ppb) across a linear range of 4–300 ppb. Specific detection of induced aggregation for Ba^2+^ can be accomplished via sulfur-containing carboxylic acid ligands. Charkova [117] synthesized spherical AgNPs (55 ± 5 nm) functionalized with 4-mercaptophenylacetic acid (MPAA) for the detection of biologically harmful barium ions (Ba^2+^) using 785 nm excitation. Ba^2+^ establishes unique “molecular bridges” with the terminal carboxyl groups of MPAA, resulting in the aggregation of nanoparticles into large clusters (>0.5 μm). SERS technology effectively detected subtle fluctuations in benzene ring breathing vibrations during aggregation, attaining an exceptionally low detection limit of 10^−15^ M (femtomolar level). This indicates a sensitivity enhancement of six orders of magnitude relative to conventional nanocolorimetric techniques, validating the practical utility of this approach in environmental toxicity assessment. The precise identification of rare earth elements (REEs) has always posed difficulties owing to their closely related chemical characteristics. Jin et al. [118] employed citrate-capped AgNPs under 488 and 532 nm excitation to develop a SERS classification platform for La^3+^ and Gd^3+^. This study examined the influence of variations in ionic 4f electron configurations (La^3+^ as 4f^0^ and Gd^3+^ as 4f^7^) on Raman scattering cross-sections. Experiments and DFT simulations revealed that the coordination of RE^3+^ with citrate molecules not only facilitated AgNP aggregation but also resulted in a notable ion dependence of the peak intensity ratio at 1065 cm^−1^ and 1315 cm^−1^ (I_1065_/I_1315_). The spectrum feature differences dependent on spin states, as illustrated in Figure 12, effectively facilitated the qualitative differentiation of these two rare earth ions.

### 4.3. Multi-Metal Ions

This section examines multifunctional SERS sensing systems that can concurrently detect or differentiate several metal ions. In real environmental monitoring, the coexistence of numerous pollutants necessitates the development of sensors with broad-spectrum responses or multi-channel recognition capabilities, which is of considerable importance.

Employing biomass molecules abundant in functional groups as reducing and capping agents facilitates the eco-friendly synthesis of nanomaterials and enhances sensors’ broad-spectrum responsiveness to various metal ions. Sharma and associates [119,120,121] have performed a series of comprehensive investigations in this domain, including microwave-assisted techniques to swiftly synthesize diverse functionalized AgNPs. Sharma et al. employed the carboxyl and hydroxyl groups on pectin chains in a pectin-based system to facilitate the aggregation of AgNPs in the presence of ions like Cr^3+^, Se^4+^, and Mn^2+^. This device accomplished dual-mode SERS and colorimetric detection by observing alterations in distinctive Raman peaks (e.g., Se^4+^ at 432 cm^−1^, Mn^2+^ at 586 cm^−1^), with a LOD for Cr^6+^ as low as 6.40 µM. The presence of such resolvable spectral signatures for different ions suggests the feasibility of parallelizing the readout to detect multiple analytes simultaneously in a single scan. Subsequently, the team broadened their research to a lignin-functionalized system, demonstrating that lignin-capped AgNPs displayed remarkable colorimetric responses to Co^2+^, Cr^3+^, and Mn^2+^, and accomplished high-sensitivity fingerprint identification of Mn^2+^ in SERS mode through signal amplification at 595 cm^−1^ (LOD 0.06 mM). In their investigation of ferulic acid, they further clarified the mechanisms differentiating the colorimetric alterations generated by Fe^2+^ from the SERS signal amplification induced by Cr^3+^ at 240 cm^−1^. This body of work illustrates that the many oxygen sites in biomass molecules can function as universal trapping agents for various metal ions, presenting considerable promise for on-site screening when integrated with portable instruments.

**Figure 13:**
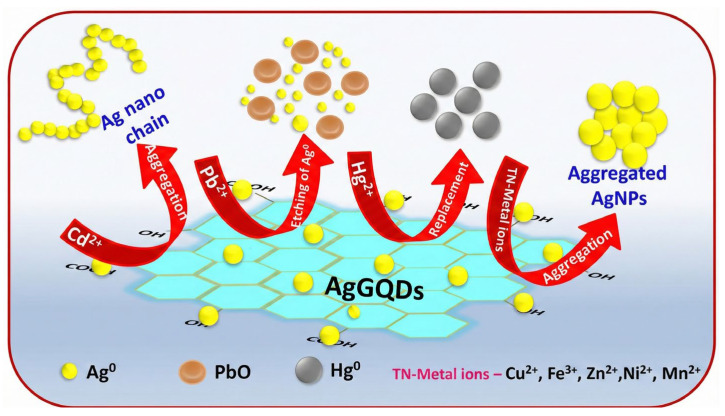
A schematic representation of the unique sensing methods for multiplex metal ion detection utilizing Ag-GQD composites, illustrating Cd^2+^-induced chain-like aggregation, Pb^2+^-initiated etching, and Hg^2+^-facilitated redox dissolution [123].

In contrast to the intricate configurations of biomass molecules, the modification with structurally specified small-molecule Raman beacons enables the development of more accurate quantitative models. Daublytė et al. [122] developed a conventional system of 4-MBA modified AgNPs for the detection of divalent metal ions (Cu^2+^, Fe^2+^, Co^2+^, Pb^2+^, etc.). The fundamental mechanism entails the creation of (-COO^−^)_2_Me^2+^ “bridging complexes” between metal ions and surface carboxyl groups. This particular chemical bridging causes substantial aggregation of AgNPs, resulting in notable SERS amplification at 1585 cm^−1^. Notably, the functionalized nanoparticles remained stable for 3 months at 8–10 °C, validating the robustness required for practical storage and use. The probe had the greatest affinity for Cu^2+^ (LOD 2.5 × 10^−7^ M) while also exhibiting a broad response to other divalent ions, hence validating the utility of carboxyl coordination-based aggregation techniques for the assessment of total divalent heavy metals. However, since this aggregation-induced readout is common to multiple ions, achieving species-specific parallel detection on a single device would necessitate the use of spatially resolved sensor arrays rather than a simple one-pot measurement. Kappen et al. [123] employed graphene quantum dots (GQDs) to modify ~20 nm AgNPs and conducted a comprehensive analysis of the distinct methods through which various metal ions induce alterations in SERS signals. As illustrated in the schematic diagram in Figure 13, the research revealed that the mechanisms of action differ significantly among ions: Cd^2+^ interacts with functional groups on the GQD surface, causing AgNPs to aggregate in chain-like formations; Pb^2+^ induces an etching effect on AgNPs and facilitates the formation of PbO; whereas Hg^2+^ instigates a redox reaction, resulting in the dissolution of Ag^0^ and the reduction/deposition of Hg^0^. This study on Ag-GQD composites surpasses basic “aggregation-enhancement” models; by linking morphological changes (e.g., sphere-to-chain transition, etching) with spectral responses, it offers a comprehensive physicochemical framework for differentiating various heavy metal ions and provides a mechanistic basis for parallelizing detection through signal-specific pattern recognition.

## 5. Dual-Mode Sensors

The previously outlined sections indicate that aggregation-based colorimetric and SERS tests originate from a shared plasmonic foundation, although they serve different analytical purposes determined by the balance of operational simplicity, cost-effectiveness, and sensitivity. Colorimetric sensing provides the unique benefit of instrument-free, visual readout, rendering it inherently appropriate for rapid, low-cost point-of-care testing (POCT); however, its use is often limited by moderate sensitivity (generally in the micromolar range) and vulnerability to false-positive results due to non-specific aggregation in complex matrices. Conversely, SERS spectroscopy provides ultra-trace detection limits and molecular-level structural fingerprinting essential for accurate quantification and interference resolution, while it requires advanced instrumentation and increased operational complexity. Therefore, depending only on one modality frequently requires a trade-off between location adaptability and analytical profundity.

The combination of colorimetric and SERS features into a unified dual-mode platform has emerged as an effective solution to address these conflicting objectives. This synergistic method integrates the swift screening ability of naked-eye detection with the thorough validation of Raman spectroscopy, employing “mutual verification” of signals to guarantee high reliability and reduce the likelihood of inaccurate readings due to environmental interferences. A foundational validation of this synergistic process was discussed in the previous chapter concerning the research of Sharma et al. [119,120,121]. Their findings have shown that ligand-mediated aggregation, even when induced by broad-spectrum biomass compounds, intrinsically produces simultaneous colorimetric and SERS readouts. This establishes a realistic foundation for the dual-mode concept, facilitating the advancement of more refined, tailored nanoprobes aimed at precisely targeting specific metal ions, as elaborated in the subsequent sections.

### 5.1. Hazardous Ions

Lead (Pb^2+^), mercury (Hg^2+^), and arsenic (As^3+^) are among the most hazardous elements posing severe threats to ecosystems and human health. Dual-mode sensing enhances detection reliability by synergizing facile colorimetric readouts with ultrasensitive SERS spectral fingerprints.

Chadha et al. [124] created a 2-thiazoline-2-thiol (TT) functionalized AuNPs sensor, enabling the differential detection of Pb^2+^ and Hg^2+^ by dual-mode analysis. Both Pb^2+^ and Hg^2+^ can induce the aggregation of AuNPs and result in a colorimetric shift (redshift) in the solution; however, their microscopic surface mechanisms differ: Hg^2+^ promotes aggregation and establishes stable Hg(TT)_2_ complexes on the gold surface, leveraging the hotspot effect for SERS signal enhancement (Signal-on), whereas Pb^2+^, while also inducing aggregation, forms Pb(TT)_2_ complexes with a weaker affinity for the gold surface, resulting in desorption and subsequent SERS signal quenching (Signal-off). The investigation via X-ray photoelectron spectroscopy (XPS) validated this competitive adsorption mechanism. Experimental results demonstrated a strong linear correlation between variations in SERS relative intensity and ion concentration within the range of 0.1 to 10 µM. The approach attained a LOD of 0.409 µM (about 0.111 ppm) for Hg^2+^ and 0.344 µM (approximately 0.096 ppm) for Pb^2+^, satisfying the sensitivity criteria for routine environmental monitoring.

Zhang et al. [125] developed Gluc/2-NT@Au@Ag core–shell probes to enhance the sensitivity for Pb^2+^ detection. The aggregation induced by Pb^2+^ leads to a significant yellow-to-dark green color change for rapid optical identification, while the resultant hotspots amplify the SERS signal of 2-NT. The colorimetric mode is designed for swift on-site screening of high-concentration materials, demonstrating a linear range from 1.0 × 10^−6^ M to 2.5 × 10^−5^ M, with a limit of detection (LOD) of 0.252 µM. The SERS mode exhibits ultrasensitive trace-level detection capabilities, showing exceptional linearity (R^2^ = 0.9919) across a concentration range of 10^−11^ M to 10^−6^ M, with a limit of detection as low as 0.185 pM. This coupling provides a unique benefit through its hierarchical detection abilities, combining the ease of visual observation for high-concentration alarms with the accuracy of SERS for trace-level quantification, thereby linking local testing with laboratory-quality analysis.

To detect As^3+^, Li et al. [126] developed a label-free approach utilizing GSH-functionalized AuNPs. The precise binding initiates aggregation, leading to a transition from wine-red to blue (LOD 0.11 ppb) and a coordinated “turn-on” SERS signal of R6G (LOD 0.14 ppb). The primary advantage of this dual-mode design is the reciprocal validation of signals; the spectroscopic fingerprint of SERS authenticates the colorimetric readout, substantially reducing false positives that commonly arise in single-mode colorimetric assays due to non-specific aggregation in intricate matrices.

### 5.2. Transition Metal and Other Metal Ions

Transition metals such as copper (Cu^2+^) frequently function as structural cofactors, attributes that have been utilized to engineer advanced dual-mode or tri-mode sensors. Guo et al. [127] devised a novel “core-satellite” assembly strategy. They employed 4-mercaptobenzoic acid (MBA)-modified AgNPs as the “core” and 4-mercaptopyridine (Mpy)-modified AuNPs as “satellites”. Cu^2+^ functioned as a metal linker, specifically connecting the carboxyl groups and pyridine nitrogen atoms, so facilitating the formation of Ag-Au heterostructures. This method accomplished visual colorimetric detection (LOD 0.032 µM) and, crucially, the high-density hotspots generated in the core-satellite gaps diminished the SERS detection limit to 0.6 pM, exhibiting a robust linear response from 1 pM to 100 µM, significantly below the EPA drinking water standard.

**Figure 14:**
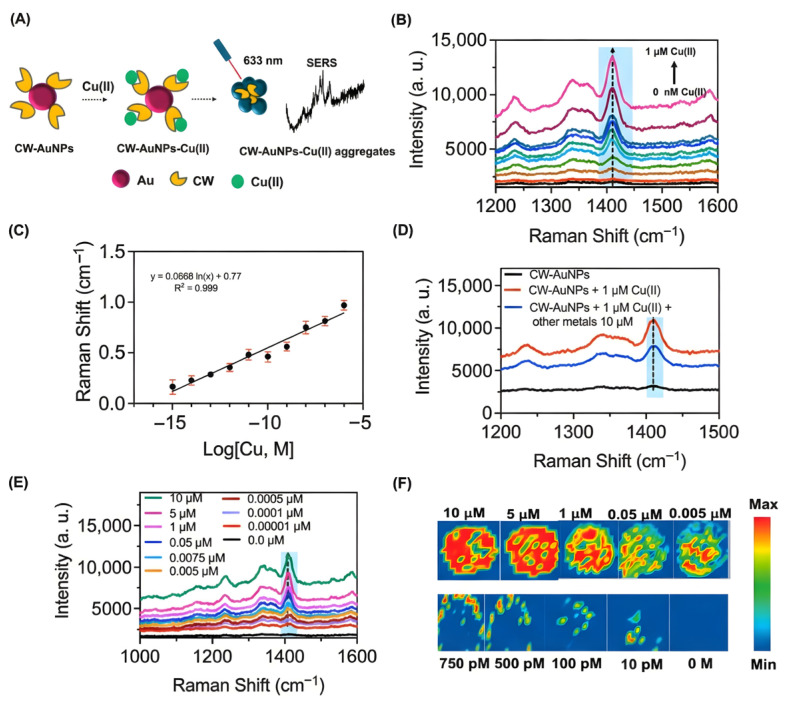
Schematic representation of the SERS sensing process utilizing Cu^2+^-induced aggregation of peptide-functionalized AuNPs and its analytical efficacy (**A**) A schematic representation of SERS-based Cu(II) detection using CW-AuNPs. (**B**) Raman spectra for CW-AuNPs (50 µg/mL) with the addition of various amounts of Cu(II). The dotted lines indicate the increase in Raman intensity at 1416 cm^−1^ upon the addition of increasing amounts of Cu(II). (**C**) Linear relationship between SERS peak intensities at 1416 cm^−1^ and Cu(II) concentrations. (**D**) Raman spectra for CW-AuNPs, CW-AuNPs + Cu(II), and CW-AuNPs + Cu(II) + various other metal ions. Studies showed high selectivity of CW-AuNPs with Cu(II), even with the addition of various other metal ions. (**E**) SERS for the dried samples of CW-AuNPs with Cu(II). Blue shaded box indicates Raman shift at 1416 cm^−1^. (**F**) Raman mapping images for the dried samples from CW-AuNPs with various concentrations of Cu(II). Excitation: 633 nm laser (6.8 mW); exposure time: 10 s [128].

Integrated colorimetric and SERS dual-mode sensing technologies leverage the corroboration of macroscopic color alterations and microscopic spectral signatures to attain extremely reliable detection of copper ions (Cu^2+^). Kumar et al. [128] introduced a high-performance dual-mode sensing approach utilizing Cystine-Tryptophan (CW) dipeptide-modified gold nanoparticles. The CW dipeptide in this system had distinctive “dual-function” properties: it acted as a particular capture probe for Cu^2+^ while employing its indole ring as an intrinsic Raman reporter. Cu^2+^ facilitated multidentate coordination with sulfur, indole nitrogen, and carbonyl oxygen within the peptide chain, resulting in the regulated aggregation of AuNPs and the formation of high-density electromagnetic hotspots (Figure 14A). The aggregation action not only altered the solution’s color from red to blue (colorimetric LOD 76 nM) but also produced a substantial linear amplification of the SERS signal at 1416 cm^−1^ (Figure 14B,C). This work additionally integrated a dry-state Raman mapping technique. Through visual examination of the signal distribution on the surface of dried droplets (Figure 14E,F), the probe’s signal homogeneity at low concentrations was effectively confirmed, finally facilitating ultrasensitive detection down to 10 pM. Moreover, portable test strips designed utilizing this technique exhibited remarkable environmental stability, affirming the practical applicability of this strategy for point-of-care testing (POCT). Zheng et al. [129] developed a tri-modal sensing platform utilizing Schiff base ligand (BAMH)-modified gold nanorods (GNRs) to augment detection dimensionality. This approach employed the robust chelation between Cu^2+^ and BAMH (Ka = 1.32 × 10^7^ M^−1^) to facilitate the self-assembly of GNRs. This action concurrently initiated three signal alterations: a redshift of the LSPR peak (colorimetric), fluorescence quenching (fluorescence mode), and a notable amplification of the SERS signal at 1940 cm^−1^. The reciprocal validation of these three modalities significantly diminished the likelihood of false positives, and this approach has been effectively utilized for detection in other domains, including ambient water samples and biological fluids (saliva, urine).

Krpetic et al. [130] examined the impact of nanoparticle size on dual-mode performance for Ni^2+^ detection with NTA/L-carnosine functionalized AuNPs. Larger nanoparticles (45 nm) optimize both colorimetric contrast and SERS intensity relative to smaller nanoparticles (14 nm) because of enhanced gap-plasmon resonances. This study emphasizes a significant benefit of dual-mode optimization: by linking macroscopic optical alterations with microscopic spectral enhancements, the system guarantees that the visual signal is directly associated with particular plasmonic coupling events, thus improving the reliability of the detection mechanism.

Gao et al. [131] developed a trimodal sensor (colorimetric/fluorescent/SERS) for Zn^2+^ utilizing Schiff base-modified AuNRs, wherein ion coordination prompts LSPR changes and enables a “turn-on” SERS response. The principal benefit of this multi-channel coupling is its self-calibration capacity, especially in biological specimens. The distinctive chemical fingerprint offered by SERS serves as a conclusive verification instrument, effectively mitigating incorrect readings that may result from matrix interferences (e.g., light scattering) impacting just the colorimetric or fluorescence channels.

## 6. Conclusions

The accurate monitoring and dynamic analysis of metal ions are crucial for ensuring ecological safety and protecting human health. Due to the constraints of conventional, bulky instrumental analysis methods in swift on-site detection applications, the advancement of detection approaches that integrate high sensitivity, affordability, and convenience has emerged as a focal point of research in analytical chemistry. Colorimetric analysis and SERS techniques utilizing the aggregation effect of Au/Ag NPs, due to their distinctive LSPR characteristics and electromagnetic field amplification mechanisms, offer effective methods for the qualitative identification and quantitative extraction of metal ions. This review comprehensively outlines methodological advancements in this domain, focusing on green synthesis techniques utilizing biomass extracts, Ligand engineering for targeted surface recognition, and sensing mechanisms that encompass oxidative etching, coordination bridging, and biomolecular conformational transitions. Moreover, the incorporation of physically assisted preparation methods, solid-phase support devices, multimodal synergistic strategies, and intelligent readout systems integrated with machine learning algorithms has significantly broadened the application potential of colorimetric and SERS techniques reliant on Au/Ag NPs aggregation in intricate environments.

Significant advancements have been achieved in enhancing detection efficacy. Notable research has accomplished “ultrasensitive” detection of trace metal ions, with LODs for certain technologies exceeding the criteria set by the World Health Organization (WHO) and pertinent environmental guidelines. Simultaneously, smart terminal-based detection devices reconcile “high sensitivity” with “on-site portability,” confirming the viability of point-of-care testing (POCT) in resource-constrained areas. The implementation of ratiometric measurements and dual-mode signal complementary techniques has provided sensors with “self-calibration” skills, significantly enhancing the reliability of analyses in complicated matrices. Nonetheless, the shift from laboratory research to effective real-world applications is hindered by significant hurdles. The colloidal stability of nanoparticles is influenced by environmental variations, such as ionic strength, while the random nature of aggregation results in heterogeneous SERS “hotspots,” undermining signal repeatability and quantitative accuracy. In addition to technical measurements, the practical implementation is additionally limited by the safety and sustainability of nanomaterials. The possible cytotoxicity of Au/Ag nanoparticles limits their direct use in in vivo biological analysis. Moreover, the absence of economical, established processes for large-scale manufacture restricts commercial feasibility, while the environmental buildup of non-degradable noble metal waste from disposable point-of-care devices presents enduring ecological hazards.

Consequently, future investigations utilizing Au/Ag nanoclusters are expected to evolve towards a multidisciplinary integration to address these impediments: (1) Formulating surface modification techniques with enhanced anti-interference properties and environmental adaptation to augment the long-term stability and specificity of probes in intricate matrices such as whole blood and wastewater; (2) Enhancing the utilization of chemometrics and artificial intelligence algorithms in spectral data processing to resolve non-linear signal responses and multiple interference challenges; (3) Establishing standardized industrial manufacturing protocols via automated or microfluidic-based synthesis to guarantee batch-to-batch consistency and reduce production costs for large-scale application; (4) Comprehensively evaluating nanotoxicological risks to validate probe safety for biological use, while developing biodegradable substrates and waste management strategies to mitigate the environmental footprint associated with noble metal accumulation; (5) Advancing the downsizing and integration of sensing platforms by amalgamating microfluidics with wearable devices to facilitate high-throughput and in situ dynamic monitoring of metal ions; and (6) Investigating nontraditional physical mechanisms, such as interfacial electron spin phenomena, to overcome obstacles in differentiating chemically analogous ions or congeners.

In conclusion, whereas aggregation-based colorimetric and SERS sensing systems demonstrate significant potential, their future efficacy depends on addressing the conflict between performance and practicality. This technology is poised to play a crucial role in environmental monitoring and biomedical diagnostics by integrating advancements in sensitivity and multimodal reliability with stringent safety assessments, standardized production, and sustainable waste management, thereby enabling a successful shift from academic research to practical applications.

## Figures and Tables

**Table 1:** Analytical efficacy of colorimetric tests for the identification of chromium ions (Cr^3+^ and Cr^6+^).

Nanostructure	Ligand	Linear Range	LOD	Method	Evaluation	Ion	Ref.
AuNPs	4-MBA	20–25 μM	5 μM	Ligand Engineering	—	Cr^3+^	[47]
ZmL-AuNPs	*Ziziphus mauritiana* extract	16–283 nM	0.48 nM	Green synthesis strategies	High-stable	Cr^3+^	[48]
NDC-AuNPs	Cystamine-functionalized NDC	10–400 nM	0.236 nM	Ligand Engineering	Ultrasensitive	Cr^3+^	[49]
AuNPs	AMT	—	1.0 μM	Ligand Engineering	—	Cr^3+^	[50]
AuNPs	ATG	0–5.0 μM	57.1 nM	Ligand Engineering	—	Cr^3+^	[51]
AuNPs & CDs	GSH	2–50 μM	0.30 μM	Multimodal sensing	Self-calibrated	Cr^3+^	[52]
Metal NPs	MMT ligands	40–128 nM	12.4 nM	Smart Readout & Algorithmic Enhancement	Sensitive & Portable	Cr^3+^	[53]
AuNPs	PMMA Microfluidic Chip	1.00–35.00 μM	0.33 μM	Smart Readout & Solid-Phase Support and Phase Transition	—	Cr^3+^	[54]
Chl-AgNPs	Chlorophyll	2–100 μM	0.62 μM	Physical Assistance & Green synthesis strategies	—	Cr^6+^	[55]
AuNPs	PVP (Plasma synthesis)	0.1–3.0 μM	0.072 μM	Physical Assistance and Post-Treatment	—	Cr^6+^	[56]
AuNPs	DPC	—	0.3 μM	Smart Readout & Special Response Mechanisms		Cr^6+^	[57]
AuNPs/AgNPs	Citrate/Na	0.96–961 μM (0.05–50 ppm)	0.44 μM	Special Response Mechanisms	Wide linear range	Cr^6+^	[58]

## Data Availability

Data are available upon request to the corresponding author.
